# Characterization of the AGR2 Interactome Uncovers New Players of Protein Disulfide Isomerase Network in Cancer Cells

**DOI:** 10.1016/j.mcpro.2021.100188

**Published:** 2021-12-18

**Authors:** Pavla Bouchalova, Lucia Sommerova, David Potesil, Andrea Martisova, Petr Lapcik, Veronika Koci, Alex Scherl, Petr Vonka, Joan Planas-Iglesias, Eric Chevet, Pavel Bouchal, Roman Hrstka

**Affiliations:** 1 Department of Biochemistry, Faculty of Science, Masaryk University, Brno, Czech Republic; 2 Regional Centre for Applied Molecular Oncology, Masaryk Memorial Cancer Institute, Brno, Czech Republic; 3 Central European Institute for Technology, Masaryk University, Brno, Czech Republic; 4 National Centre for Biomolecular Research, Faculty of Science, Masaryk University, Brno, Czech Republic; 5 Department of Human Protein Sciences, Faculty of Medicine, University of Geneva, Geneva, Switzerland; 6 Loschmidt Laboratories, Department of Experimental Biology and RECETOX, Faculty of Science, Masaryk University, Brno, Czech Republic; 7 International Clinical Research Center, St Anne's University Hospital Brno, Brno, Czech Republic; 8 INSERM U1242, “Chemistry, Oncogenesis, Stress, Signaling”, Université Rennes 1, Rennes, France

**Keywords:** anterior gradient protein 2, protein–protein interactions, protein disulfide isomerase, mass spectrometry, secretory pathway, Ab, antibody, ADT3, arogenate dehydratase 3, AGR2, anterior gradient 2, BiP, binding immunoglobulin protein, CAM, carbamidomethyl, DMSO, dimethyl sulfoxide, DSP, dithiobis[succinimidylpropionate], ENPL, endoplasmin, ER, endoplasmic reticulum, FA, formic acid, FDR, false discovery rate, FLNB, filamin-B, GLU2B, glucosidase 2 subunit beta, GO, Gene Ontology, GRP78, glucose-regulated protein, 78 kDa, GSEA, gene set enrichment analysis, HNRPU, heterogeneous nuclear ribonucleoprotein U, HSPA5, heat shock 70 kDa protein 5, IP, immunoprecipitation, IT, ion trap, LFQ, label-free quantification, log2FC, log2 fold change, LTQ, linear trap quadrupole, MS, mass spectrometry, NC, negative control, NP-40, Nonidet P-40, OT, Orbitrap, PBS-T, Tween-20 in PBS, PCCA, propionyl-CoA carboxylase alpha chain, PD, pull down, PDB, Protein Data Bank, PDI, protein disulfide isomerase, PLA, proximity ligation assay, PPI, protein–protein interaction, PYC, pyruvate carboxylase, RT, room temperature, RPN1, ribophorin 1, SILAC, stable isotope labeling with amino acids in cell culture, THG, thapsigargin, TUN, tunicamycin, UT, urea–Tris

## Abstract

Anterior gradient 2 (AGR2) is an endoplasmic reticulum (ER)-resident protein disulfide isomerase (PDI) known to be overexpressed in many human epithelial cancers and is involved in cell migration, cellular transformation, angiogenesis, and metastasis. This protein inhibits the activity of the tumor suppressor p53, and its expression levels can be used to predict cancer patient outcome. However, the precise network of AGR2-interacting partners and clients remains to be fully characterized. Herein, we used label-free quantification and also stable isotope labeling with amino acids in cell culture–based LC–MS/MS analyses to identify proteins interacting with AGR2. Functional annotation confirmed that AGR2 and its interaction partners are associated with processes in the ER that maintain intracellular metabolic homeostasis and participate in the unfolded protein response, including those associated with changes in cellular metabolism, energy, and redox states in response to ER stress. As a proof of concept, the interaction between AGR2 and PDIA3, another ER-resident PDI, was studied in more detail. Pathway analysis revealed that AGR2 and PDIA3 play roles in protein folding in ER, including post-translational modification and in cellular response to stress. We confirmed the AGR2–PDIA3 complex formation in cancer cells, which was enhanced in response to ER stress. Accordingly, molecular docking characterized potential quaternary structure of this complex; however, it remains to be elucidated whether AGR2 rather contributes to PDIA3 maturation in ER, the complex directly acts in cellular signaling, or mediates AGR2 secretion. Our study provides a comprehensive insight into the protein–protein interaction network of AGR2 by identifying functionally relevant proteins and related cellular and biochemical pathways associated with the role of AGR2 in cancer cells.

## Highlights

• LC–MS/MS analysis of AGR2-interacting proteins in T47D and H1299 cells.
• About 15 overlapping AGR2 interactors, including PDIA3 and PDIA6, were identified in both cell lines.
• PDI family members represent the key part of the network.
• AGR2–PDIA3 interaction is even stronger under ER stress.
• AGR2–PDIA3 complex formation supports extracellular secretion of AGR2.

## Graphical abstract

**Figure:**
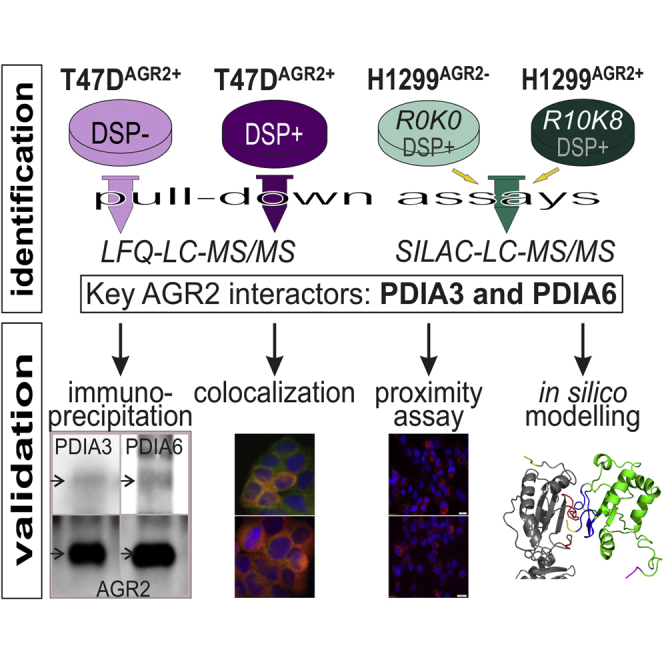

## In Brief

Quantitative LC-MS/MS analysis of AGR2 interactome has identified 15 potential partners in both T47D cells and H1299 cells stably transfected with AGR2. The most interesting partners, PDIA3 and PDIA6, belong to the protein disulfide isomerase family. Stronger PDIA3 interaction with AGR2 under ER stress further supports the existence of PDI reactive network in the cells.

Protein–protein interactions (PPIs) are essential for the correct structure and function of a vast majority of protein complexes. Alterations in PPIs may significantly contribute to the regulation of key biological processes, such as cell growth, proliferation, and cellular homeostasis (1). Thus, identification and analysis of the physical interactions between various proteins are crucial for uncovering physiological protein functions and understanding the molecular mechanisms responsible for human diseases. Mass spectrometry (MS) represents the technology of choice to sensitively and reliably identify and map PPIs in a variety of biological samples (2).

Anterior gradient 2 (AGR2), the human homolog of *Xenopus laevis*–secreted protein XAG-2, is a member of the protein disulfide isomerase (PDI) family abundantly expressed in the endoplasmic reticulum (ER) (3). The ER plays an important role in the biosynthesis, processing, and transport of proteins and lipids in eukaryotic cells. AGR2 as an ER-resident protein is suggested to play an essential role in the protein quality control by interacting with nascent polypeptides, forming disulfide bonds, and thus contributing to the maintenance of ER homeostasis (4, 5). Importantly, AGR2 is also induced by ER stress *via* activation of the activating transcription factor 6 and inositol-requiring enzyme 1 arm of the unfolded protein response in order to protect cells from the stress caused by misfolded and/or unfolded proteins (6). AGR2 was reported to be overexpressed in many epithelial tumors (7), and its secretion was proposed to serve as an important disease biomarker (8, 9). Elevated levels of AGR2 were shown to significantly contribute to aggressive tumor growth, survival, and metastasis development (10, 11, 12).

However, little is known about AGR2-interacting partners in tumor cells. The first attempt using yeast two-hybrid screening identified glycosylphosphatidylinositol-anchored metastasis-associated protein C4.4a and extracellular dystroglycan 1 (dystrophin-associated glycoprotein 1) as prominent AGR2-binding partners (13). Later on, AGR2 was shown as an essential mediator for the production of intestinal mucus with the assumption that a cysteine residue within the AGR2 thioredoxin-like domain forms mixed disulfide bonds with mucin 2, indicating a direct role for AGR2 in mucin processing (14). ER mammalian protein–protein interaction trap was recently published, allowing specific detection of AGR2 PPIs in the ER (15). Transmembrane emp24 domain containing protein 2, as a major regulator of AGR2 dimerization, was identified by this approach.

Search for AGR2-interacting partners within the Biological General Repository for Interaction Dataset database (https://thebiogrid.org/115802/summary/homo-sapiens/agr2.html; June 22, 2021) revealed 948 proteins including C4.4a and dystroglycan 1 that physically interact with AGR2 (16). However, less is known about the function of the AGR2 in protein–protein complexes. Therefore, we used reversible crosslinking followed by pull down (PD) of AGR2 complexes and high-resolution LC–MS/MS to identify proteins interacting with AGR2 in order to assign the role of found complexes to respective signaling pathways in cancer cells.

## Experimental Procedures

### Cell Culture and Treatment

Human cancer cell lines T47D, A549, and H1299 were maintained in high glucose Dulbecco's modified Eagle's medium, supplemented with 10% fetal bovine serum, 300 μg/ml l-glutamine, 100 IU/ml penicillin, and 100 μg/ml streptomycin at 37 °C in a humidified atmosphere with 5% CO_2_. Throughout the duration of all experiments, cells were free from mycoplasma. For the induction of ER stress, the cells were treated with different ER inducers such as thapsigargin (THG; 100 nM), tunicamycin (TUN; 1 μg/ml), and DTT (0.5 mM) or maintained in the serum-free medium for 16 h. The Flp-In System (Thermo Fisher Scientific, Inc) was used to generate H1299-LZ4 cells containing a single integrated Flp recombination target site. The coding sequence of the human *AGR2* gene was stably inserted into this site using Flp recombinase–mediated site-specific DNA recombination to give H1299-LZ4-AGR2 cell line. H1299-LZ4 and H1299-LZ4-AGR2 (here and thereafter H1299 and H1299-AGR2) cells used for MS experiments were maintained in “stable isotope labeling with amino acids in cell culture” (SILAC) Dulbecco's modified Eagle's medium containing unlabeled (R0K0; light) or labeled lysine and arginine (R10K8; heavy), respectively (Dundee Cell Biosciences), in three replicates each. Transfection was carried out using 2 μg of plasmid or 50 pmol of siRNA per million cells. To silence *AGR2* or *PDIA3* gene expression, cells were transiently transfected with siRNAs against AGR2, PDIA3, or untargeted siRNA serving as a control (Dharmacon/Thermo Fisher Scientific) using nucleofection in Amaxa Nucleofector II (Lonza). pcDNA3-AGR2 plasmid was used to express AGR2 in transiently transfected cells.

### Protein Crosslinking and Extraction

Cells were grown up to 80% confluence and washed three times with PBS (0.1 M phosphate, 0.15 M NaCl, and pH 7.2) directly on plates. 0.6 mM dithiobis[succinimidylpropionate] (DSP) (Thermo Fisher Scientific) in 1.4% dimethyl sulfoxide (DMSO) in PBS (control: DMSO in PBS) was applied for 30 min at room temperature (RT) (10 ml of solution per 15 cm dish). The crosslinking reaction was stopped by the addition of Tris at pH 7.5 (final concentration of 10 mM) for 15 min. Cells were washed twice with PBS and lysed in lysis buffer (150 mM NaCl, 50 mM Tris [pH 8], 50 mM NaF, 5 mM EDTA [pH 8], 1% Nonidet P-40 [NP-40], 1% protease inhibitor cocktail [Sigma–Aldrich], and 1 mM PMSF), incubated on ice with shaking for 30 min, then sonicated by 30 × 0.1 s pulses and 1 s pauses on maximum power in Vibra-Cell sonicator (Sonics & Materials, Inc) and centrifuged 14,000*g*/30 min/4 °C. Reducing agent and detergent compatible protein assay (Bio-Rad) was used to determine protein concentration.

### PD of AGR2 Using Biotinylated Peptides

**Fig. 1:**
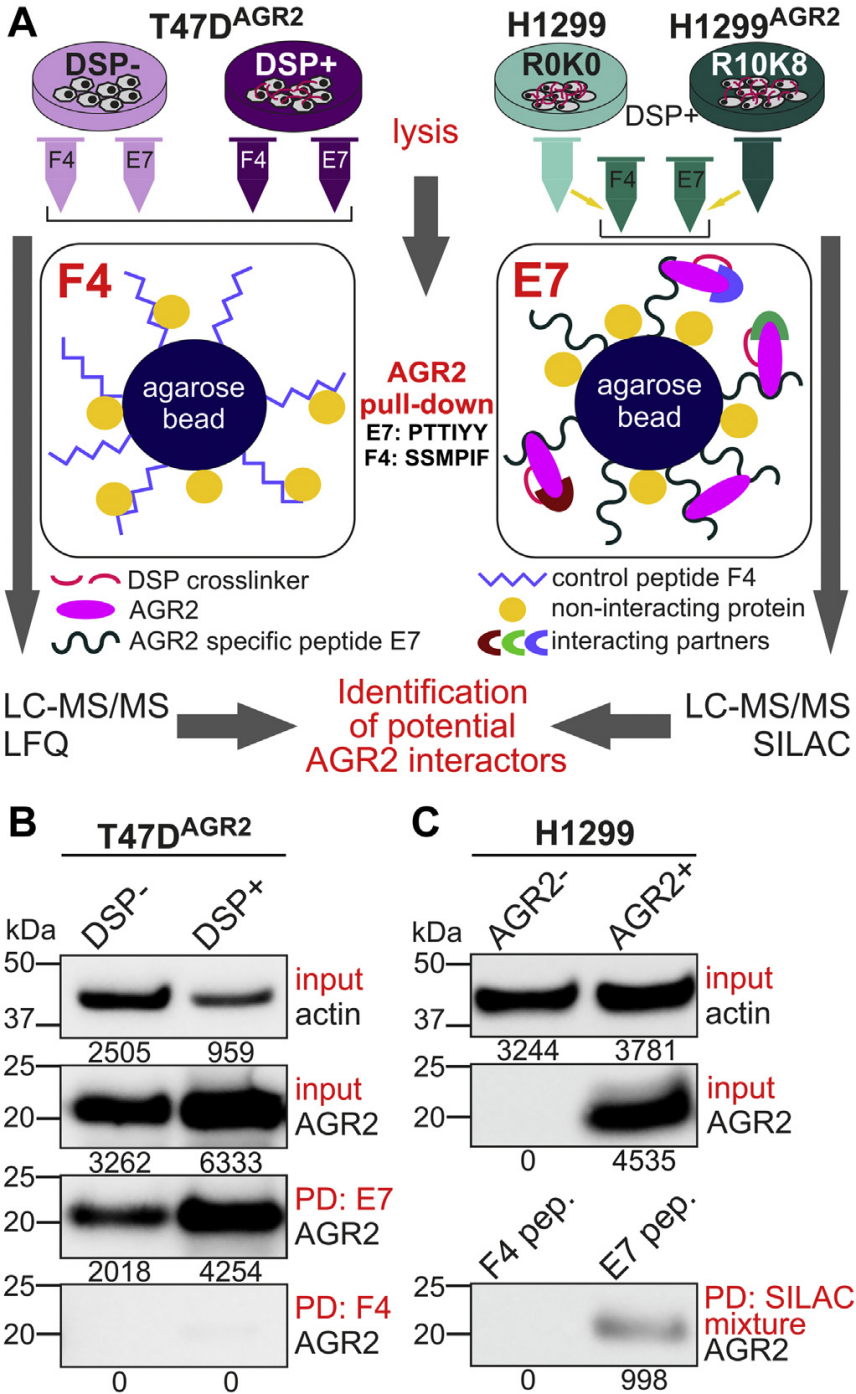
**Workflow of AGR2 pull-down (PD) methods and protein-level analysis.***A,* two different cell lines were used for AGR2 PD. Breast cancer cell line T47D, of which endogenously expressed AGR2 protein underwent DSP crosslinking and PD with AGR2-specific E7 peptide and untargeted control peptide F4. Cells without DSP treatment served as a control. Corresponding MS data were quantified using label-free quantification (LFQ). H1299 lung carcinoma cell line was stably transfected with vector carrying coding sequence of AGR2. AGR2 positive clone was labeled with heavy (R10K8) SILAC medium, whereas parental H1299 served as a control and was maintained in light (R0K0) SILAC medium. These cells underwent DSP crosslinking, and lysates were, according to total protein content, equally mixed into one sample, and PD was done with E7 (AGR2 specific) and F4 (control) peptides again. MS data were quantified using SILAC approach. *B* and *C,* protein levels of AGR2 were detected in all input samples (20 μg of total protein per well) as well as in eluted proteins (10 μl of eluates per well) using immunoblotting with anti-AGR2 and antiactin antibodies, which served as a loading control. Numbers under the bands represent integral absorbance (INT∗mm^2^∗10^3^) obtained by Quantity One software (Bio-Rad). AGR2, anterior gradient 2; DSP, dithiobis[succinimidylpropionate]; MS, mass spectrometry; SILAC, stable isotope labeling with amino acids in cell culture.

200 μl of streptavidin–agarose suspension (Sigma–Aldrich) was washed in 0.1% Tween-20 in PBS (PBS-T) and labeled with 4.5 μl of biotinylated 6aa-aptamers (5 mg/ml in DMSO) called E7 (AGR2-targeting, PTTIYY) and F4 (untargeted control) overnight on rotating wheel at 4 °C and washed 6× with PBS-T (17). These aptamer-containing mixtures were mixed with 400 μg of total protein lysate from T47D cells or mixture of 200 μg of R0K0 H1299 and 200 μg of R10K8 H1299-AGR2 lysates and incubated overnight at 4 °C on a rotating wheel and then washed 6× with PBS-T (see Fig. 1*A* for a schematic overview). 50 μl of 0.1 M glycine (pH 2.5) were used for elution of interacting proteins for 10 min at RT. The eluates were neutralized with 5 μl of 1.5 M Tris at pH 8.8.

### Trypsin Digestion of Potentially AGR2-interacting Proteins

Trypsin digestion was performed using filter-aided sample preparation with several modifications (18). The whole eluates were added onto Vivacon 500 ultrafiltration spin columns (10 kDa membrane cutoff; Sartorius Stedim Biotech) with 200 μl of urea–Tris (UT) buffer (8 M urea in 0.1 M Tris at pH 8.5). The columns were centrifuged at 14,000*g*/15 min/20 °C. 100 μl of UT buffer was then added onto the columns followed by the addition of 40 μl 100 mM Tris(2-carboxyethyl)phosphine, mixed, and left in thermomixer (Eppendorf) for 30 min/600 rpm/37 °C to reach a complete reduction of proteins. The samples were then centrifuged at 14,000*g*/15 min/20 °C. Subsequently, 100 μl of UT buffer and 20 μl of 300 mM iodoacetamide were added onto the columns and mixed. The samples were first alkylated for 1 min/600 rpm/25 °C in thermomixer and then 20 min in the dark without shaking, followed by centrifugation at 14,000*g*/15 min/20 °C. The columns were washed twice with 100 μl of 50 mM ammonium bicarbonate and centrifuged for 14,000*g*/20 min/20 °C. The digestion was performed by the addition of 2 μl of 0.25 μg/μl trypsin (SCIEX) in 50 μl of 50 mM NH_4_HCO_3_ followed by incubation for 12 h at 37 °C in a wet chamber. The digests were collected by centrifugation at 14,000*g*/15 min/20 °C. The columns were then washed again with 50 μl of 0.5 M NaCl by centrifugation at 14,000*g*/15 min/20 °C, and the digests were desalted as follows.

### Peptide Desalting Prior to LC–MS/MS

C18 Silica MicroSpin columns (NestGroup, Inc) were used to desalt the peptides prior to MS analysis (19). The columns were first washed twice with 200 μl of 0.1% TFA in acetonitrile at 300*g*/3 min/RT, followed by two washes with 200 μl of 0.1% TFA in water at 300*g*/3 min/RT, then left to hydrate for 15 min at RT, and centrifuged at 300*g*/3 min/RT. The digests were loaded onto the columns and centrifuged at 500*g*/3 min/RT. The columns were then washed three times with 200 μl of 0.1% TFA in water and centrifuged at 500*g*/3 min/RT. The elution was performed by adding 200 μl of 0.1% TFA in 80% acetonitrile and centrifugation at 500*g*/3 min/RT, followed by 200 μl of 0.1% TFA in acetonitrile and centrifugation under the same conditions. Both eluates were pooled, filtered through 0.22 μm polyvinylidene fluoride microfilter (Merck KGaA—Millipore), and dried under vacuum.

### LC–MS/MS Analysis With Label-free Quantification

LC–MS/MS analyses with label-free quantification (LFQ) were performed on NanoAcquity LC system (Waters) on-line connected to Linear Trap Quadrupole (LTQ) Orbitrap (OT) Velos MS system (Thermo Electron). Peptides were trapped on a home-made 5 μm 200 Å Magic C18 AQ (Michrom Bioresources) 0.1 × 2 mm precolumn and separated on a home-made 5 μm 100 Å Magic C18 AQ (Michrom Bioresources) 0.75 × 150 mm column with a gravity-pulled emitter. The analytical separation was run for 65 min using a gradient of water/formic acid (FA) 99.9%/0.1% (solvent A) and CH_3_CN/FA 99.9%/0.1% (solvent B). The gradient was run as follows: 0 to 1 min 95% A and 5% B, then to 65% A and 35% B at 55 min, and 20% A and 80% B at 65 min at a flow rate of 220 nl·min^−1^. For MS survey scans, the OT resolution was set to 60,000, and the ion population was set to 5 × 10^5^ with an *m/z* window from 400 to 2000. A maximum of eight precursors was selected for collision-induced dissociation in the LTQ. For MS/MS in the LTQ, the ion population was set to 1 × 10^4^ (isolation width of 2 *m/z*), and dynamic exclusion was enabled for 45 s. The normalized collision energy was set to 35%. The samples were run in four technical replicates (injections).

### LC–MS/MS Analysis With SILAC Quantitation

LC–MS/MS analyses with SILAC quantitation were performed on a different system consisting of RSLCNano on-line connected to OT Elite MS system (Thermo Fisher Scientific). Peptides were trapped on a 3.5 μm X-Bridge BEH 130 C18 sorbent (Waters) 0.1 × 30 mm precolumn and separated on a 2 μm Acclaim Pepmap100 C18 (Thermo Fisher Scientific) 0.75 × 250 mm column directly connected to the Nanospray Flex Ion Source (Thermo Fisher Scientific). The analytical separation was run for 95 min using a gradient of water/FA 99.9%/0.1% (solvent A) and CH_3_CN/methanol/trifluoroethanol/FA 59.9%/30%/10%/0.1% (solvent B). The gradient was run as follows: 1 to 2 min from 99% A and 1% B to 98% A and 2% B, then to 89% A and 11% B at 30 min, then to 75% A and 25% B at 60 min, then to 55% A and 45% B at 90 min, and 5% A and 95% B at 95 min at a flow rate of 300 nl·min^−1^. For MS survey scans, the OT resolution was set to 240,000, and the ion population was set to 1 × 10^6^ with an *m/z* window from 350 to 1700. A maximum of top 10 precursors was selected for collision-induced dissociation in the ion trap (IT). For MS/MS in the IT, the ion population was set to 1 × 10^4^ (isolation width of 2 *m/z*), and the dynamic exclusion was enabled for 45 s. The normalized collision energy was set to 35%. The samples were run in two technical replicates (injections).

### MS Data Analysis

Protein identification and quantification was performed in MaxQuant 1.5.7.4 (www.maxquant.org) using Andromeda database search algorithm. The data analysis parameters were as follows: *database:* UniProt/SwissProt human database 2017_03 downloaded from http://www.uniprot.org (March 30, 2017) with 20,183 protein sequences (complemented by database of common protein contaminants according to the standard Andromeda settings); *enzyme name*: trypsin (cleaving polypeptides at the carboxyl side of lysine or arginine); *maximum missed cleavage sites* 2; *taxonomy*: *Homo sapiens*. *Decoy database search:* peptide sequence match false discovery rate (FDR) of 0.01, protein FDR of 0.01, and site FDR of 0.01. *Tolerances:* 20 ppm/4.5 ppm (first search/main search) peptide tolerance and 0.5 Da IT MS/MS fragment match tolerance. *Modifications:* dynamic (variable): oxidation (M), acetyl (protein N-term), carbamidomethyl (CAM)-thiopropanoyl (protein N-term), and CAM-thiopropanoyl (K). Static (fixed): CAM (C). *LFQ:* fast LFQ, minimum ratio count of 2. *SILAC quantification:* multiplicity 2, maximum labeled AAs 3, light—no labels, heavy—Arg10, Lys8 labels.

### AGR2 Interactome Analysis

Gene set enrichment analysis (GSEA) in GSEA Java desktop application (http://software.broadinstitute.org/gsea/downloads.jsp) was conducted using the preranked list (according to protein log2 fold change [log2FC]) of 478 AGR2-interacting proteins with log2FC >0 quantified using LFQ in T47D E7 cells with DSP treatment to T47D E7 cells without DSP treatment, and 215 AGR2-interacting proteins with log2FC >0 quantified using SILAC in AGR2 stably transfected heavy H1299 E7 cells to heavy H1299 F4 cells, to find enriched pathways separately, with *a priori* defined pathways from BioCarta (https://cgap.nci.nih.gov/Pathways/BioCarta_Pathways) and Reactome (https://reactome.org/). We used default settings, except that we decreased the minimal size of a gene set to 1. Top 20 protein–protein interacting partners of AGR2 according to log2FC with *q* value <0.05 identified in T47D cells using LFQ and AGR2 stably transfected H1299 cells using SILAC quantitation were visualized separately using Cytoscape Java desktop application, version 3.7.0 (https://cytoscape.org/) (20). Log2FC was used as target node attribute and *q* value as edge attribute. In parallel, only interacting proteins showing significant log2FC (log2FC >0; *q* < 0.05) were selected and compared between T47D (151 proteins) and H1299 (26 proteins) cells. The proteins overlapping in both cell lines were subsequently analyzed by the Cytoscape 3.7.0 using ClueGo Plugin, version 2.5.5 (21, 22) and ConsensusPathDB-human (http://cpdb.molgen.mpg.de/CPDB) that contains information from Gene Ontology (GO) database and Kyoto Encyclopedia of Genes and Genomes pathways databases and was developed to analyze protein–protein, genetic, metabolic, signaling, gene regulatory, and drug–target interactions. The default settings were used, and level 3 categories of GO terms were selected with *p* value <0.001 cutoff.

### Molecular Docking

The two peptides E7 (SGSGPTTIYY) and F4 (SGSGSSMPIF) used for immunoprecipitation (IP) (17, 23) underwent the molecular docking with AGR2. PEP-FOLD3 framework (https://bioserv.rpbs.univ-paris-diderot.fr/services/PEP-FOLD3/; (24, 25, 26)) was used for *de novo* prediction of peptide structure models. Number of simulations was 200. Generated models were sorted by their sum of Optimized Potential for Efficient structure Prediction values (the coarse-grained energy of PEP-FOLD). The predicted model with the best value of sum of Optimized Potential for Efficient structure Prediction energy was chosen for each peptide, and these models were used for further docking study by HADDOCK docking server.

The protein–peptide docking was performed in triplicates using the HADDOCK 2.4 web server (https://wenmr.science.uu.nl/haddock2.4/; (27)). The structures of AGR2 protein (Protein Data Bank [PDB] ID: 2LNS; (28)) and AGR3 protein (PDB ID: 3PH9; (29)) were downloaded from the PDB (https://www.rcsb.org/; (30)). The active residues were specified in case of AGR2 protein according to available literature (AA131–135; (23)) and in case of AGR3 protein according to alignment of protein sequences from UniProt server (AGR2 protein: https://www.uniprot.org/uniprot/O95994; AGR3 protein: https://www.uniprot.org/uniprot/Q8TD06) by on-line BLAST web interface (AA122–126; (31, 32)). Passive residues were defined automatically around the active residues. Only chain A of each protein structure was used for docking. Data were converted in highly ambiguous interaction restraints to drive docking with the protein monomer and the aformentioned described peptides. Default settings were used for docking parameters. Briefly, number of structures for rigid body docking was 10,000, number of structures for semiflexible refinement was 400, number of structures for the explicit solvent refinement was 400, fraction of common contact was used as clustering method, RMSD cutoff for clustering was 0.60, and minimum cluster size was 4. Resulted structures were determined according to interactions describing in the literature and predominantly interface energy terms.

Models of AGR2–PDIA3 complex were generated by *ab initio* docking on GalaxyHeteromer web server (http://galaxy.seoklab.org/cgi-bin/submit.cgi?type=HETEROMER; (33)) and ClusPro web server (34) using default parameters. The structures of AGR2 protein (PDB ID: 2LNS; (28)) and PDIA3 protein (PDB ID: 3F8U; (35)) were downloaded from the PDB (https://www.rcsb.org/; (30)). Both AGR2 monomer and dimer were used for docking. The obtained models were ranked by the GalaxyTongDock_A cluster size and the GalaxyTongDock_A score in the case of GalaxyHeteromer or by cluster size and cluster centroid energy (corresponding to the balanced model) in the case of ClusPro. PyMOL software (The PyMOL Molecular Graphics System, version 2.0; Schrödinger, LLC) and PDBsum web server (http://www.ebi.ac.uk/thornton-srv/databases/pdbsum/Generate.html; (36)) were used for visual inspection of models of AGR2–PDIA3 heterocomplexes to identify most probable interaction interface according to the presence or the absence of interaction motifs of both proteins. Based on these criteria, one model of heterocomplex with AGR2 monomer and one model of heterocomplex with AGR2 dimer were chosen for further structure refinement by Rosetta online server (ROSIE; https://rosie.graylab.jhu.edu/; (37)). Rosetta docking protocol was used with option “docking_local_refine.” About 25 structures of each protein complex were generated. The resulting structures were determined according to known interactions and interface energy terms. Finally, an independent blind-docking–based binding energy prediction was done for both AGR2 monomer–PDIA3 and AGR2 dimer–PDIA3 using BADock (38) from crystallographic tertiary structures. BADock is a method based on the intermolecular funnel-like energy landscape theory that exploits all docking solutions (considering all good and bad energies) to make its predictions.

### Western Blotting

For the preparation of cell lysates, the cells were twice washed with ice-cold PBS and lysed in lysis buffer (120 mM NaCl, 50 mM Tris–HCl [pH 7.2], 1% NP-40 [v/v], 1 mM EDTA, 6 mM EGTA, 6 mg/ml sodium pyrophosphate, 1× protease inhibitor cocktail, and 1× phosphatase inhibitor cocktail [both Sigma–Aldrich]) for 30 min on ice. Lysates were cleared by centrifugation at 13,000 rpm/30 min/4 °C. Proteins were separated by Mops SDS-PAGE (39) and transferred to nitrocellulose membrane using the Tetra Cell-Blot (Bio-Rad) with 1× blotting buffer (20 mM Tris, 150 mM glycine, 20% methanol, and pH 8.3). Proteins were detected using rabbit anti-AGR2 (1:1000; K31 in house), mouse anti-AGR2 (1:500; Abnova), mouse anti-AGR2 (1:1000; AG3 4.1 in house), mouse anti-PDIA3, rabbit anti-PDIA6 (both 1:1000; Abcam), mouse anti-β-actin (1:1000; Sigma–Aldrich) as a loading control, and species-specific secondary horseradish peroxidase–coupled antibodies (1:1000; Dako). Densitometry analysis was performed using Quantity One software (Bio-Rad).

### Isolation of Cellular Fractions

Cytosolic fraction and membrane-bound (ER) cellular fractions were isolated as previously described (40) with a few modifications to the protocol. Briefly, cells were detached from culture plates by trypsin, and pellets were washed twice with cold PBS. The pellets were resuspended in buffer 1 (50 mM Hepes [pH 7.4], 150 mM NaCl, and 25 and 40 μg/ml digitonin for A549 and T47D, respectively) and incubated for 30 min at 4 °C with rotation. After the incubation, samples were centrifuged at 2000*g*/5 min/4 °C. The supernatants were transferred into clean Eppendorf tubes and marked as cytosolic fraction. The pellets were resuspended in buffer 2 (50 mM Hepes [pH 7.4], 150 mM NaCl, and 1% NP-40) and incubated for 30 min on ice. Afterward, the samples were centrifuged at 7600*g*/10 min/4 °C, and the supernatants were again transferred and marked as ER (membrane-bound fraction containing ER).

### IP

For IP experiments, cells were extracted using lysis buffer supplemented with a complete protease inhibitor cocktail (Sigma–Aldrich). Cell lysates (200 μg of total protein) were incubated with the corresponding antibody (Ab) in concentration of 1 μg/ml overnight at 4 °C with gentle agitation. Complexes were separated by incubation with Protein G Sepharose 4 Fast Flow beads (GE Healthcare) at 4 °C for 2 h, followed by two washes in lysis buffer and one wash with PBS. Samples were eluted using 2× Laemmli buffer, boiled, and loaded into gel.

### Determination of Extracellular Proteins

The medium from cells was collected after 16 h treatment and subjected to centrifugation at 14,000 rpm/10 min/4 °C. Ice-cold acetone was added to culture media at a ratio of 1:4 and incubated at −20 °C overnight. The protein precipitate was collected by centrifugation at 14,000 rpm/10 min/4 °C. The protein pellet was dried by heating to 95 °C for 10 min and resuspended with 2× Laemmli buffer and separated under denaturing conditions by SDS-PAGE (39). Membranes were incubated with primary antibodies and detected as described previously.

### Immunofluorescence

Cells were seeded on sterile cover slides (Thermo Fisher Scientific) and grown in the respective cultivation medium. Then, the cells were washed with PBS and fixed with 4% formaldehyde (Sigma–Aldrich) diluted in PBS for 20 min at RT. After incubation, cells were washed with PBS for 5 min and permeabilized with 0.2% Triton X-100 (Sigma–Aldrich) diluted in PBS for 5 min at RT. Permeabilized cells were washed again with PBS and blocked for 30 min at RT in 3% bovine serum albumin (Sigma–Aldrich) diluted in PBS-T. Afterward, cells were incubated with primary antibodies diluted in 3% bovine serum albumin in PBS-T in 4 °C overnight. AGR2-specific (Abnova) and PDIA3-specific (Abcam) antibodies were diluted 1:500, and Ab against PDIA6 (Invitrogen) was diluted 1:250. On the following day, the cells were washed thrice with PBS and then incubated with Alexa Fluor 488 goat antimouse IgG and Alexa Fluor 594 goat anti-rabbit IgG (both Abcam) together with Hoechst 33342 (Sigma–Aldrich) for 60 min at RT in the dark. Coverslips were washed three times with PBS, then once with distilled water, and mounted with VECTASHIELD mounting medium (Vector Laboratories). Slide images were acquired on Olympus BX41 (Olympus). Colocalization was determined according to the adapted protocol by Moser *et al.* (41) using the colocalization plug-in of Fiji software (https://imagej.net/software/fiji/; (42)). For each experimental condition, Pearson's correlation was determined for at least six wide-field pictures (≥100 labeled cells) in three independent experiments. Both positive controls and negative controls (NCs) were included in the study to verify the accuracy of colocalization analysis (supplemental Fig. S1). As NCs, A549 cells with AGR2 gene knockout were used (mean Pearson's correlation coefficient = 0.16). In contrast, cells labeled with two different fluorochromes for AGR2 (obtained by anti-AGR2 mouse Ab from Abnova and anti-AGR2 rabbit Ab from Abcam followed by fluorochrome-conjugated secondary antibodies from Abcam mentioned previously) served as the positive control for determination of colocalization (mean Pearson's correlation coefficient = 0.93).

### Proximity Ligation Assay

For proximity ligation assay (PLA), the cells were seeded on cover slips and fixed either with (i) 4% formaldehyde for 10 min at 37 °C and then washed twice with 0.02% PBS-T and permeabilized using 0.5% Triton X-100 in PBS for 5 to 10 min at RT or (ii) chilled methanol:acetone 1:1 for 10 min and dried at RT for 3 h (in the case of T47D cell line). Afterward, the cells were incubated with blocking buffer (Sigma–Aldrich—Duolink) for 1 h at 37 °C in a wet chamber. Primary antibodies against AGR2 (1:250; Abnova), PDIA3 (1:500; Abcam), and PDIA6 (1:250; Novus Biologicals) were then incubated in Ab diluent (Duolink) at 4 °C overnight. The PLA was performed with the Duolink *In Situ* Red Kit Mouse/Rabbit (Sigma–Aldrich) according to the manufacturer's instructions. Anti-mouse MINUS and anti-rabbit PLUS PLA probes (Sigma–Aldrich) were used. Coverslips were mounted with Vectashield mounting medium, and images were acquired by Olympus BX41 microscope using a 40× objective. Images were analyzed with CellSens software (Olympus).

### Experimental Design and Statistical Rationale

The workflow of the proteomics experiment is shown in Fig. 1*A*. Identification of AGR2-interacting partners was performed with T47D breast cancer cells, which naturally expresses a high level of AGR2 protein. The cells were crosslinked with DSP, whereas control cells were not treated. The isolated proteins from DSP+/DSP− samples underwent PD with anti-AGR2–specific peptide E7 or with control nonspecific peptide F4. LC–MS/MS analysis was run four times (injections) per sample (technical replicates). In parallel, independent analysis of AGR2-negative H1299 lung carcinoma cells stably transfected with the AGR2-expressing vector was done using SILAC technique. H1299 control cells grew in SILAC light (R0K0) medium, whereas H1299-AGR2 cells grew in SILAC heavy (R10K8) medium; both cell cultures underwent DSP crosslinking. After cell lysis, the equal amounts of total proteins from both cell populations were mixed together, and PDs with anti-AGR2–specific E7 or control F4 peptides were performed. LC–MS/MS has been run twice per sample (technical replicates).

Statistical analysis of MS data was performed in Perseus 1.5.8.5 (www.maxquant.org). Proteins identified by the search against a decoy database and only by a modification site were removed prior to analysis. The data were log2-transformed, and missing values were replaced by a normal distribution. Protein fold changes were calculated from LFQ intensities or from intensities of heavy and light protein form (SILAC). Data were statistically analyzed using two-sample *t* test with permutation-based FDR correction; protein level changes with *q* < 0.05 were considered statistically significant.

From the eight proteins overlapping between the lists of AGR2 potential interactors (log2FC > 2; *q* <0.05) in both cell lines with cellular localization relevant for AGR2 (ER; supplemental Table S1), two proteins sharing the same protein family (PDIA3 and PDIA6) were selected for the validation. A panel of molecular and *in silico* methods of different principles (IP, immunofluorescence, PLA, and molecular docking) was used to validate the interaction between AGR2 and identified partners and to find the biological role of the interactions.

## Results

### Identification of AGR2-interacting Partners

To identify proteins interacting with AGR2 in T47D cells, we used 0.6 mM DSP serving as an *in vivo* intracellular protein crosslinker. DSP-free cells were used as a parallel control. AGR2 complexes with interacting proteins were targeted using AGR2-specific peptide E7, whereas F4 peptide served as an NC (Fig. 1, *A* and *B*). Direct binding of E7 peptide with AGR2 has been identified by Murray *et al.* (17) and confirmed with peptide mapping by hydrogen/deuterium exchange (23). In line with these findings, the model of AGR2 protein in complex with the E7 peptide was generated by molecular docking (supplemental Fig. S2). Briefly, HADDOCK clustered 315 to 333 structures in 22 to 24 clusters representing in between 78 and 83.75% of the analyzed docking solutions across the triplicates in the case of AGR2 protein in complex with E7 peptide, whereas F4 peptide only rendered 234 to 247 structures in 24 to 30 clusters representing 58 to 61% of the analyzed docking solutions across the triplicates (supplemental Figs. S2 and S3). These results show a clear trend for a more cohesive docking of E7 peptide compared with that of F4, which might be interpreted as a hint for better binding as well. We selected for further investigation a model of the best docking solution for each of the peptides to AGR2. Such a model consisted of a representative from the top ranked cluster from the corresponding docking experiment that conformed to previous experimental observations (17, 43): the N terminus of the peptide should be free, and the C terminus bound to AGR2 (supplemental Fig. S4). Predominantly, amino acids 131 to 135 in AGR2 represent the specific binding site for E7 peptide (23). Interestingly, these amino acids are in AGR2 protein sequence in close proximity to amino acids 150 to 156, which create a disordered region unique for AGR protein family (28). F4 peptide was used as NC according to previous work by Murray *et al.* (17). The rationale for F4 peptide utilization is also supported by HADDOCK-predicted models showing clear decrease in electrostatic energy of F4 peptide compared with E7 peptide on interaction interface within amino acids 131 to 135 (supplemental Figs. S2, *B* and *C*, *G* and *H*, and *L* and *M*; S3, *B* and *C*, *G* and *H*, and, *L* and *M*; S4, *A* and *C*). Remarkably, PDBsum predicts that AGR2 forms a larger number of hydrogen bonds with E7 peptide, 4, than with F4, only 1 (supplemental Fig. S4, *B* and *D*).

**Fig. 2:**
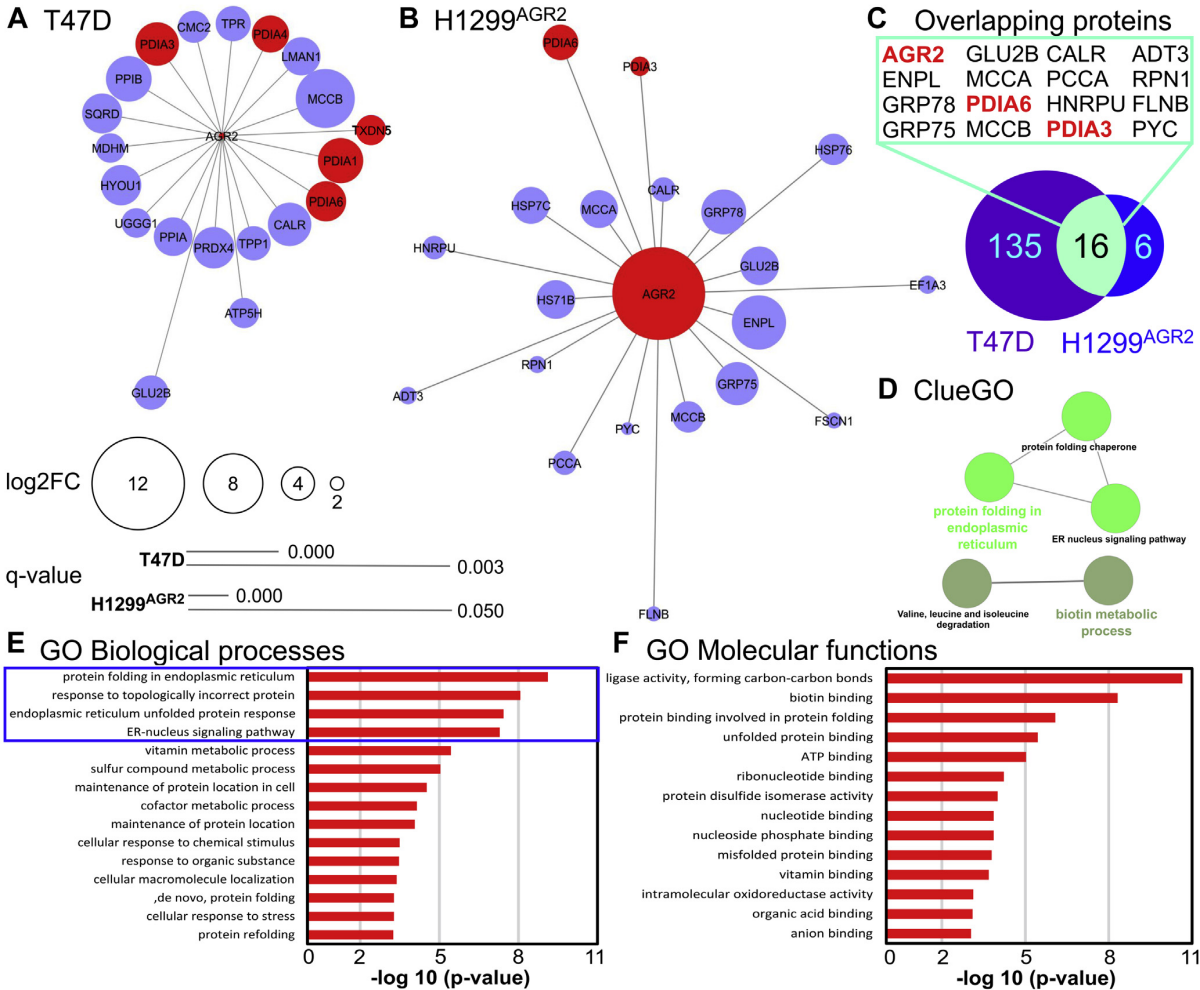
**Annotation of AGR2 protein–protein complexes in relation to cell signaling.** Top 20 protein–protein interacting partners of AGR2 (log2FC >0; *q* < 0.05) identified in (*A*) T47D cells using LFQ and (*B*) H1299–AGR2 cells using SILAC quantitation. See supplemental Data Files S1 and S2 for source data for *A* and *B*, respectively. Proteins in *red* are members of the PDI family, proteins in *blue* are not members. *C,* 151 and 22 proteins (log2FC >0; *q* < 0.05) were identified as AGR2-interacting partners in T47D and H1299–AGR2 cells, respectively. Comparison of these proteins between cell lines has selected 16 overlapping proteins (supplemental Table S1). PDI members are highlighted in *red*. *D,* Cytoscape ClueGO analysis (based on GO and KEGG pathway databases) of 16 overlapping proteins (supplemental Table S1) has revealed a clear connection of proteins to the ER and ER processes. Enriched GO and KEGG terms are represented by nodes, and protein overlap between the terms is displayed by edges. Similarly, analysis of 16 overlapping proteins (supplemental Table S1) with ConsensusPathDB-human tool with GO and KEGG databases clearly showed strong connection to (*E*) biological processes running in ER highlighted in *blue box* and to (*F*) molecular functions associated with protein translation and folding in ER, all pathways with *p* value <0.001 as determined by ConsensusPathDB-human tool. AGR2, anterior gradient 2; ER, endoplasmic reticulum; GO, Gene Ontology; KEGG, Kyoto Encyclopedia of Genes and Genomes; LFQ, label-free quantitation; log2FC, log2 fold change; PDI, protein disulfide isomerase; SILAC, stable isotope labeling with amino acids in cell culture.

After incubation of cell lysate with peptide, the crosslinker was reduced, and the trypsin-digested PD eluates were analyzed by LC–MS/MS (supplemental Data File S1). Among these, five of the top 20 potential interactors (according to log2FC) belong to the PDI family: PDIA1, PDIA3, PDIA6, PDIA4, and thioredoxin domain–containing protein 5 (Table 1 and Fig. 2*A*). To verify such identified AGR2-interacting proteins in an independent cell model and by a different approach, we grew H1299 cells stably transfected to produce AGR2 (H1299-AGR2) in heavy (R10K8) SILAC medium, simultaneously with control H1299 cells (not expressing AGR2) grown in light (R0K0) SILAC medium (Fig. 1, *A* and *C*). All cells were treated with 0.6 mM DSP. AGR2-specific E7 peptide was used to target AGR2 protein in a complex with its potential interacting partners, whereas F4 peptide served as an NC. The trypsin-digested PD eluates were analyzed using LC–MS/MS. SILAC-based quantification showed that AGR2, PDIA3, and PDIA6 proteins were significantly more abundant in PD eluates of E7-targeted lysates from DSP-crosslinked H1299-AGR2 positive cells, compared with PD eluates of F4-targeted lysates from the same cells (Table 2; supplemental Data File S2; Fig. 2*B*). We also identified the previously described AGR2-interacting partner ER chaperone binding immunoglobulin protein (BiP), also known as glucose-regulated protein, 78 kDa (GRP78)/BiP or heat shock 70 kDa protein 5 (HSPA5) (28), in both cell lines. These data verify protein complex development of AGR2 with PDIA3, PDIA6, and several other proteins in independent cell lines and using a different MS quantification approach.

**Table 1:** Top 20 proteins more abundant (according to log2FC) in E7 native eluates of DSP-crosslinked T47D compared with control T47D cell

Rank (FC)	Unique peptides	Unique sequence coverage (%)	Molecular weight (kDa)	*Q* value	Score	Student's *t* test *p* value	Student's *t* test *q* value	Log2 LFQ intensity DSP−	Stddev LFQ intensity DSP−	Log2 LFQ intensity DSP+	Stddev LFQ intensity DSP+	Log2FC DSP+ *versus* DSP−	Majority protein IDs
1	25	60.7	61.332	0	323.31	6.26E-06	0.0000	19.780	1.082	27.740	0.063	7.961	sp|Q9HCC0|MCCB_HUMAN
2	11	48.1	23.742	0	94.608	1.47E-05	0.0000	19.650	0.991	25.956	0.080	6.306	sp|P23284|PPIB_HUMAN
**3**	**15**	**37.4**	**57.116**	**0**	**232.81**	**0.000100594**	**0.0002**	**20.008**	**1.365**	**26.240**	**0.154**	**6.232**	**sp|P07237|PDIA1_HUMAN**
4	11	37.6	48.141	0	121.56	1.80E-05	0.0000	19.191	0.979	25.220	0.098	6.029	sp|P27797|CALR_HUMAN
**5**	**18**	**45.9**	**56.782**	**0**	**234.53**	**7.10E-06**	**0.0000**	**20.547**	**0.603**	**26.556**	**0.579**	**6.009**	**sp|P30101|PDIA3_HUMAN**
6	7	36.5	30.54	0	77.968	1.93E-05	0.0000	20.098	0.955	25.890	0.048	5.792	sp|Q13162|PRDX4_HUMAN
7	20	25.2	111.33	0	212.68	4.53E-07	0.0000	19.551	0.484	25.204	0.093	5.653	sp|Q9Y4L1|HYOU1_HUMAN
**8**	**10**	**35**	**48.121**	**0**	**113.21**	**5.91E-05**	**0.0002**	**19.310**	**1.122**	**24.917**	**0.085**	**5.607**	**sp|Q15084|PDIA6_HUMAN**
9	10	24.9	49.96	0	142.79	8.18E-05	0.0002	19.271	1.187	24.867	0.083	5.596	sp|Q9Y6N5|SQRD_HUMAN
10	8	63	18.012	0	80.46	3.83E-07	0.0000	19.655	0.440	25.090	0.139	5.435	sp|P62937|PPIA_HUMAN
11	6	17.3	57.548	0	42.052	5.59E-06	0.0000	19.059	0.708	24.443	0.125	5.384	sp|P49257|LMAN1_HUMAN
**12**	**10**	**18.3**	**72.932**	**0**	**87.717**	**6.98E-07**	**0.0000**	**19.767**	**0.445**	**24.994**	**0.207**	**5.227**	**sp|P13667|PDIA4_HUMAN**
13	13	7.3	267.29	0	155.42	1.80E-06	0.0000	19.192	0.545	24.195	0.085	5.003	sp|P12270|TPR_HUMAN
14	9	20.1	59.425	0	99.857	0.001046798	0.0023	20.788	1.662	25.697	0.042	4.909	sp|P14314|GLU2B_HUMAN
15	3	9.8	61.247	0	27.436	1.90E-06	0.0000	19.229	0.407	24.127	0.361	4.898	sp|O14773|TPP1_HUMAN
16	9	15.6	74.175	0	133.49	4.30E-06	0.0000	18.956	0.573	23.554	0.131	4.598	sp|Q9UJS0|CMC2_HUMAN
17	10	73.9	18.491	0	82.565	0.001077225	0.0022	19.650	1.464	24.040	0.299	4.390	sp|O75947|ATP5H_HUMAN
**18**	**6**	**16.7**	**47.628**	**0**	**41.273**	**0.000152509**	**0.0006**	**19.411**	**1.010**	**23.760**	**0.212**	**4.349**	**sp|Q8NBS9|TXND5_HUMAN**
19	9	6.2	177.19	0	68.64	0.000143058	0.0002	19.286	1.007	23.603	0.109	4.317	sp|Q9NYU2|UGGG1_HUMAN
20	8	31.4	35.503	0	101.79	9.16E-06	0.0000	19.628	0.623	23.936	0.061	4.308	sp|P40926|MDHM_HUMAN
…													
**351**	**22**	**73.7**	**19.979**	**0**	**323.31**	**0.005562899**	**0.0089**	**32.243**	**0.166**	**32.619**	**0.066**	**0.376**	**sp|O95994|AGR2_HUMAN**

The rows in bold are depicted PDI family members. In addition, AGR2 data (in the last row) were less significant as AGR2 binds similarly to E7 peptide from both types of native cell lysates. *q* Values are after FDR correction.

**Table 2:** AGR2, PDIA3, and PDIA6 protein levels in E7 (AGR2-specific peptide) native eluates from DSP-crosslinked H1299 cells stably transfected with AGR2 gene compared with F4 (control) native eluates from the same cells

Unique peptides	Unique sequence coverage (%)	Molecular weight (kDa)	*Q* value	Score	Student's *t* test *p* value	Student's *t* test *q* value	Log2 intensity heavy E7	Stddev log2 intensity heavy E7	Log2 intensity heavy F4	Stddev log2 intensity heavy F4	Log2FC heavy E7 *versus* heavy F4	Majority protein IDs
6	46.3	19.979	0	323.31	1.578E-12	0.0000	27.606	0.281	15.092	0.683	12.514	sp|O95994|AGR2_HUMAN
2	4.6	56.782	0	11.799	0.003810	0.0307	17.982	1.632	15.295	0.651	2.687	sp|P30101|PDIA3_HUMAN
6	20.9	48.121	0	80.899	0.008953	0.0478	19.848	3.505	15.038	0.993	4.810	sp|Q15084|PDIA6_HUMAN

Potential explanation of the difference in MS hit numbers found between T47D and H1299 cells would be the origin of the cell lines: breast cancer cell line T47D *versus* H1299 lung carcinoma cell line and/or the nature of the AGR2 protein expression: very high endogenous level in T47D in comparison to artificial AGR2 expression in H1299 cells stably transfected with the vector carrying *AGR2* coding sequence (Fig. 1, *B* and *C*). Semiquantitative densitometric analysis of T47D-DSP+ lysate (input sample) showed 5.5 times higher normalized signal of AGR2 endogenous level compared with H1299-AGR2 DSP+ lysate. Accordingly, the detected signal of pull-downed AGR2 protein was 4.26 times stronger for T47D cell lysate in comparison to the H1299 mixture sample. Thus, different levels of AGR2 protein may significantly affect the numbers of proteins identified in particular samples (1058 proteins in T47D to 245 proteins in H1299), indicating that the difference is not biased by the MS approach and quantitation used.

### Functional Annotation of AGR2 Interaction Network

All identified proteins with log2FC >0 in both cell lines were subjected to GSEA to visualize potential molecular relationships of these proteins to BioCarta pathways and Reactome. The most important cellular processes and signaling pathways associated with AGR2-interacting partners are listed in supplemental Table S2. These data indicate independently for both cellular models that proteins interacting with AGR2 are involved predominantly in processes situated into the ER, which contribute to maintaining of intracellular metabolic homeostasis and/or to responses to ER stress such as the unfolded protein response, changes in cellular metabolism, energy, and redox state. The top 20 proteins interacting with AGR2 identified in T47D cells (Fig. 2*A*) and H1299-AGR2 cells (Fig. 2*B*) were visualized using Cytoscape. In total, 16 overlapping proteins (log2FC >0; *q* < 0.05) were identified in both cell lines to form complexes with AGR2 (Fig. 2*C*), including ER residential (AGR2, endoplasmin [ENPL], GRP78, glucosidase 2 subunit beta [GLU2B], PDIA6, PDIA3, calreticulin [CALR], and ribophorin 1 [RPN1]), mitochondrial (GRP75, MCCA and MCCB, propionyl-CoA carboxylase alpha chain [PCCA], arogenate dehydratase 3 [ADT3], and pyruvate carboxylase [PYC]), cytoskeletal (filamin-B [FLNB]), and nuclear (heterogeneous nuclear ribonucleoprotein U [HNRPU]) proteins (for details, see supplemental Table S1). These proteins were subjected to functional annotation by GO terms. Analysis of biological functions has revealed that proteins interacting with AGR2 play roles predominantly in the protein folding in the ER and in the regulation of specific mitochondrial metabolic pathways as demonstrated by the analysis of enriched Kyoto Encyclopedia of Genes and Genomes pathways using Cytoscape ClueGO tool (Fig. 2*D*). The analysis of biological processes in ConsensusPathDB-human database revealed prevalence of the processes responsible for proper protein folding, correct protein subcellular localization, and achievement and maintenance of intracellular homeostasis (Fig. 2*E*). The top four processes take place in the ER. The analysis of GO molecular functions of these proteins (Fig. 2*F*) shows mostly chaperone and oxidoreductase functions.

### Validation of Selected Proteins Interacting With AGR2

**Fig. 3:**
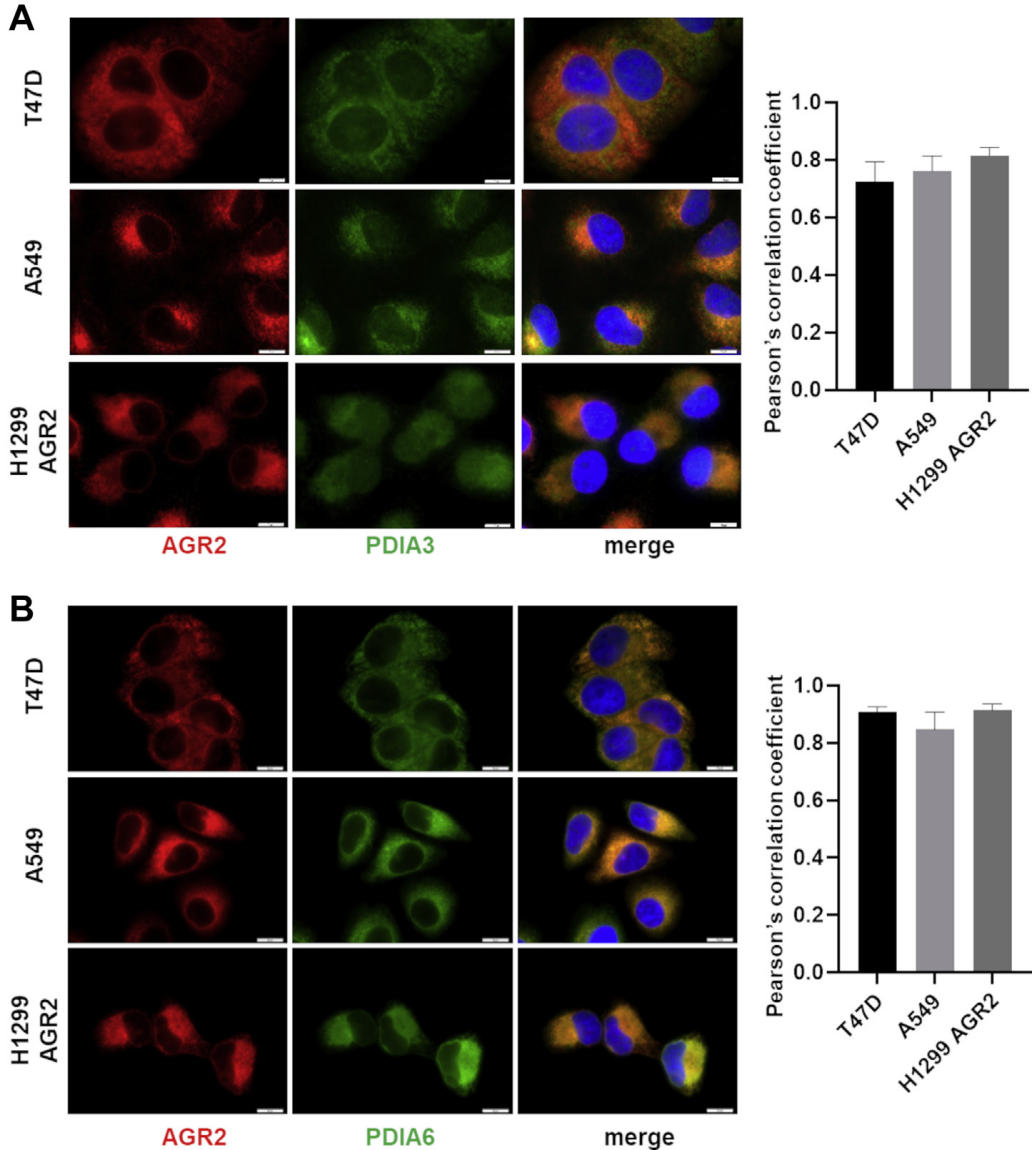
**Immunofluorescence microscopy of PDIA3 and PDIA6 in relation to AGR2.** Immunofluorescence staining of AGR2 (*red*) in parallel with (*A*) PDIA3 (*green*) and (*B*) PDIA6 (*green*). Merged images show colocalization of these proteins indicating the presence of AGR2–PDIA3 and AGR2–PDIA6 complexes. Nucleic staining (*blue*) was done by Hoechst 33342. The scale bar represents 10 μm. Colocalization of fluorescence signals was determined by Pearson's correlation coefficient (graphs on the *right side*). See supplemental Fig. S1 for a positive colocalization control. AGR2, anterior gradient 2; PDIA3, protein disulfide isomerase A3.

**Fig. 4:**
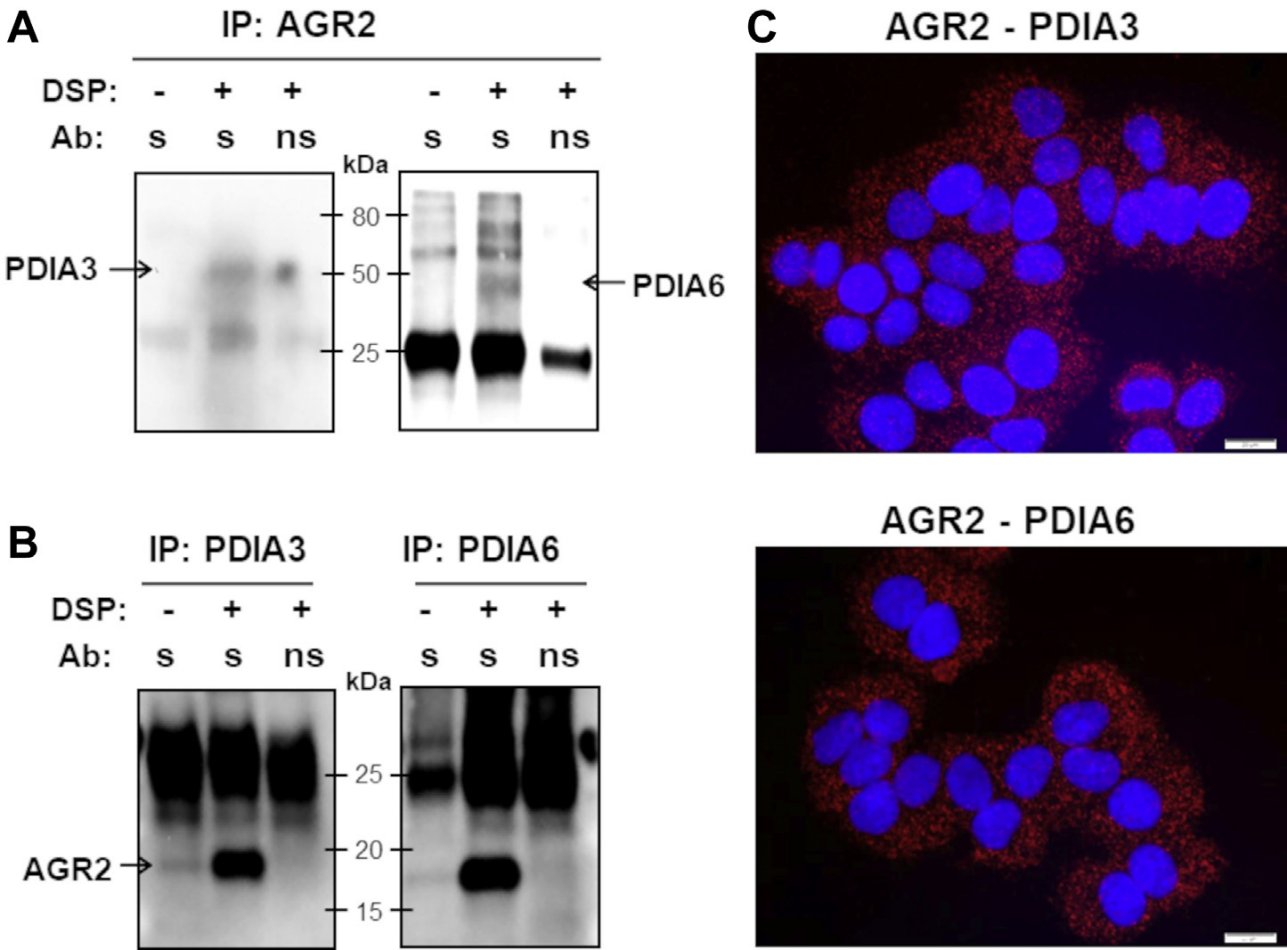
**Validation of PDIA3 and PDIA6 as AGR2-interacting partners.** The combined procedures of IP and SDS-PAGE were used in complex protein mixtures from T47D cells either exposed or unexposed to DSP in order to (*A*) precipitate AGR2 by specific antibody (s); (*B*) precipitate PDIA3 (*left part*) and PDIA6 (*right part*) by specific antibodies (s). Nonspecific antibody (ns) served as a negative control (*third line*). *C,* PLA images of complexes AGR2–PDIA3/6: *red signals* emerge only when proteins are closely localized. Nucleic staining (*blue*) was done by DAPI. The scale bar represents 20 μm. See supplemental Fig. S5 for corresponding PLA results in H1299 and A549 cell lines. AGR2, anterior gradient 2; DAPI, 4′,6-diamidino-2-phenylindole; IP, immunoprecipitation; PDIA, protein disulfide isomerase A; PLA, proximity ligation assay.

PDIs, PDIA3 and PDIA6, identified and verified by both MS approaches in both cell lines were selected to confirm their interaction with AGR2. To visualize colocalization of AGR2 with PDIA3 and PDIA6, respectively, we used fluorescence microscopy. Since T47D cell line has strong endogenous expression of AGR2 and H1299 cells were genetically engineered to produce AGR2, we introduced A549 cells as a new cell model showing moderate endogenous expression of both AGR2 and PDIA3 proteins. Indeed, similar to T47D and H1299-AGR2 cells, PDIA3 and AGR2 were located in the same area in A549 cells. Clear colocalization of AGR2 with both PDIA3 and PDIA6 is also confirmed in all cell lines by correlation coefficients >0.7 for PDIA3 and >0.8 for PDIA6 as demonstrated by the accompanying charts (Fig. 3). Encouraged by these data, we prepared cell lysates from T47D cells that were incubated with specific antibodies recognizing AGR2 or PDIA3/6. Following PD assay, IP confirmed the interaction of AGR2 with both PDIA3 and PDIA6 (Fig. 4*A*) and in reverse (Fig. 4*B*). Because of the fact that IP does not reflect intracellular spatial arrangement, PLA *in situ* was used to confirm development of AGR2 complexes with PDIA3 and PDIA6 (Fig. 4*C* and supplemental Fig. S5). The presence of both AGR2–PDIA3 and AGR2–PDIA6 complexes was observed inside the tumor cells, both transfected with expression plasmid for AGR2 (H1299-AGR2 cells; supplemental Fig. S5, *A–D*), and producing AGR2 endogenously (T47D cells, Fig. 4*C* and A549 cells, supplemental Fig. S5, *E* and *F*).

### The Role of AGR2–PDIA3 Complex in Cancer Cells

**Fig. 5:**
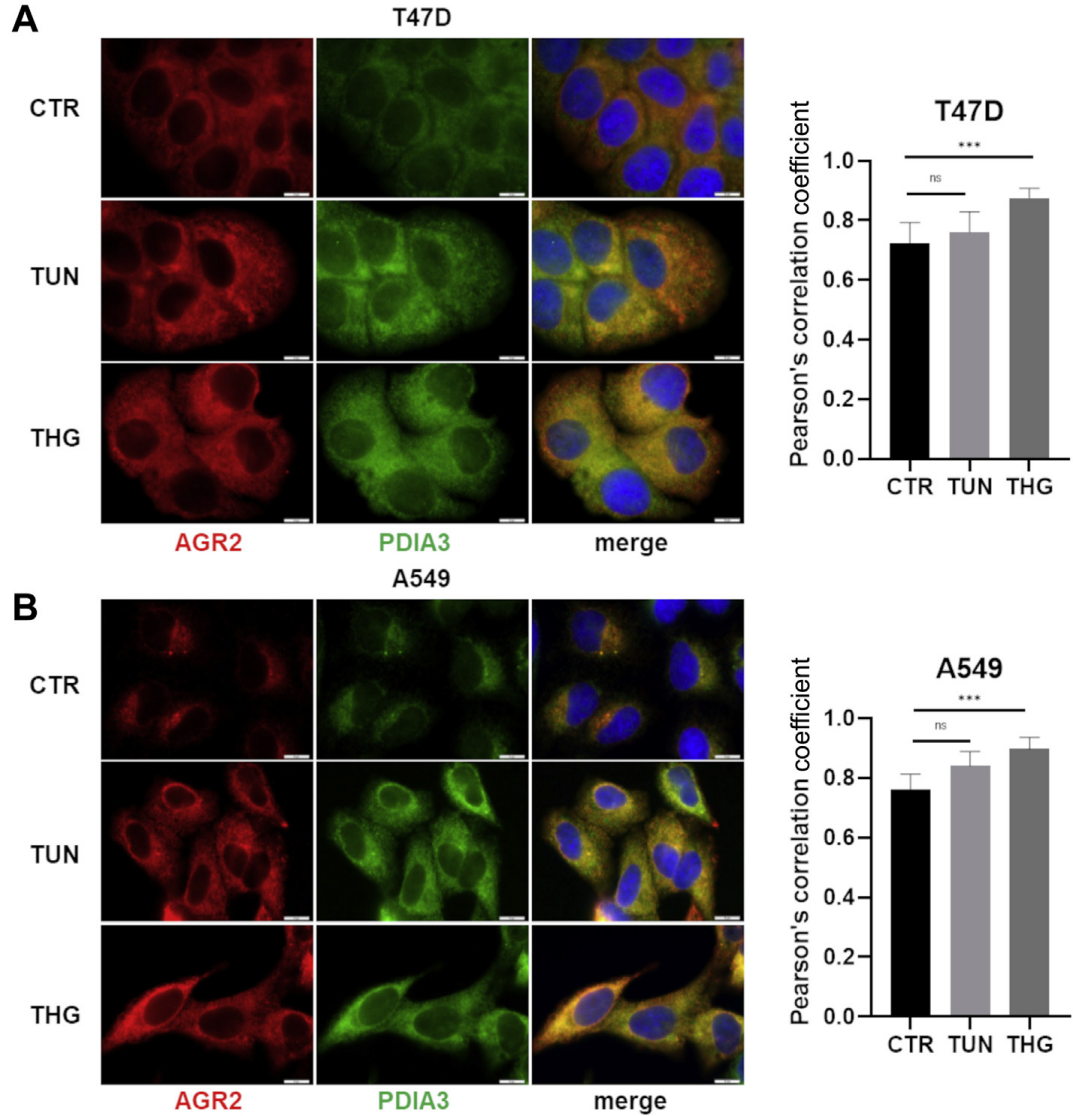
**The effect of ER inducers on AGR2–PDIA3 complex formation.** Changes in subcellular localization of AGR2–PDIA3 complex in response to tunicamycin (TUN) and thapsigargin (THG) in comparison with untreated (control, CTR) (*A*) T47D and (*B*) A549 cells were analyzed using immunofluorescence staining for AGR2 (*green*), PDIA3 (*red*), and nucleus by DAPI (*blue*). The scale bar represents 10 μm. Colocalization of fluorescence signals was determined by Pearson's correlation coefficient, nonparametric one-way ANOVA (Kruskal–Wallis test with Dunn correction) test was used to calculate the statistical significance, ∗∗∗*p* ≤ 0.001. AGR2, anterior gradient 2; DAPI, 4′,6-diamidino-2-phenylindole; ER, endoplasmic reticulum; ns, nonsignificant; PDIA3, protein disulfide isomerase A3.

The effect of ER stress on AGR2–PDIA3 location was analyzed by fluorescence microscopy that confirmed strong colocalization of these two proteins in perinuclear space in T47D. In parallel, we also observed clear disperse delocalization of the AGR2–PDIA3 complex into the cytoplasm in response to both TUN and THG (Fig. 5*A*). Interestingly, mean Pearson's correlation coefficient increased after ER stress from 0.72 for untreated control cells to 0.88 for cells exposed to THG (Fig. 5*A*) indicating that AGR2–PDIA3 colocalization is strengthened in response to ER stress. Almost the same effect was observed for A549 cells, in which the induction of ER stress enforced the colocalization of AGR2 and PDIA3 as well (Fig. 5*B*).

**Fig. 6:**
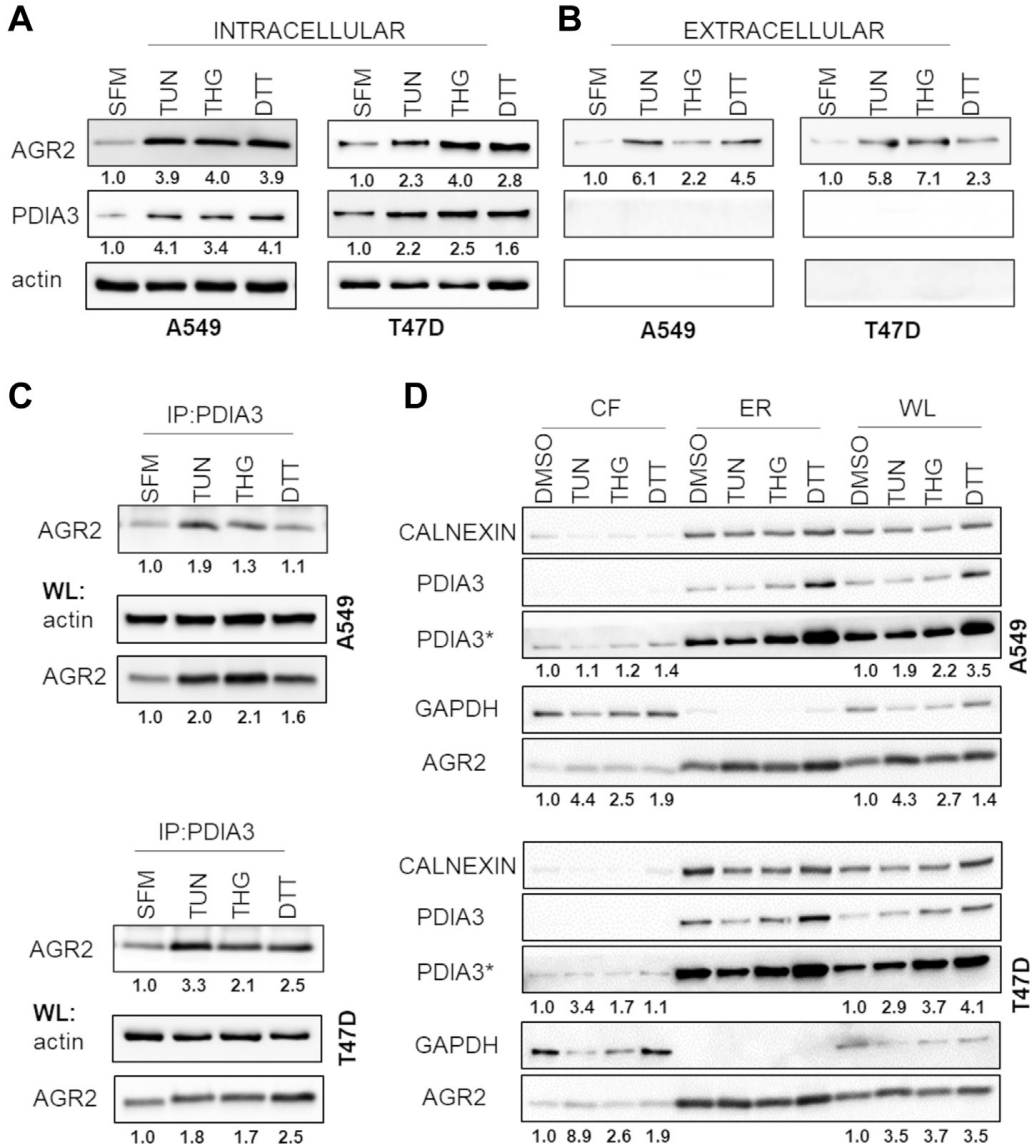
**ER stress induces complex formation followed by enhanced secretion of AGR2.** Immunochemical analysis of (*A*) intracellular and (*B*) extracellular AGR2 and PDIA3 in response to induction of ER stress. The numbers *under the boxes* represent relative fold changes in absorbance reflecting protein levels normalized on intracellular actin density of treated cells in relation to serum-starved cells (serum-free media [SFM]). *C,* IP of AGR2–PDIA3 complexes followed by SDS-PAGE in cells exposed to different inducers of ER stress. *D,* subcellular protein fractionation of several ER-resident proteins in A549 and T47D cells treated with ER stress inducers. The numbers *under the boxes* represent fold changes in absorbance reflecting protein levels normalized on GAPDH density of treated cells in relation to DMSO-exposed cells. PDIA3∗ represents the same experiment however, with prolonged exposition time to show redistribution of PDIA3 to the cytosol. Each experiment was performed at least three times. Average fold changes along with standard deviations are shown in supplemental Fig. S6. AGR2, anterior gradient 2; CF, cytosolic fraction; DMSO, dimethyl sulfoxide; ER, membrane bound fraction containing endoplasmic reticulum; IP, immunoprecipitation; PDIA3, protein disulfide isomerase A3; WL, whole lysate.

Following data from fluorescence microscopy, we investigated the expression of AGR2 and PDIA3 in relation to their ability to develop complexes under different conditions evoking ER stress. Maintaining cells in serum-free media was associated with negligible changes in both AGR2 and PDIA3 intracellular levels (data not shown). On the other hand, TUN, THG, and DTT induced the expression of intracellular AGR2 and PDIA3 in both A549 and T47D cells (Fig. 6*A* and supplemental Fig. 6*A*). Since AGR2 could also be secreted, extracellular levels of both AGR2 and PDIA3 were determined. Interestingly, a significant increase in AGR2 secretion was observed in response to all ER stress inducers, but the most when exposed to TUN, indicating that blocking of N-linked glycosylation associated with unfolded protein response may activate secretory pathway(s) leading to secretion of AGR2 from tumor cells (Fig. 6*B* and supplemental Fig. 6*B*). On the other hand, the secretion of PDIA3 was not detected in either of the cell lines. These data indicate that ER stress induces secretion of AGR2 but to what extent it most probably depends on the type of stress and the cellular context, including PDI interaction network, and remains shrouded in mystery. Interestingly, IP revealed that in response to ER stress and predominantly to TUN treatment, the complex of PDIA3 with AGR2 is developed to a greater extent compared with untreated cells (Fig. 6*C* and supplemental Fig. 6*C*). These data confirm the enhanced formation of AGR2–PDIA3 complex in response to ER stress but does not explain disperse delocalization observed with fluorescence microscopy. Therefore, we focused on evolutionary conserved ER surveillance mechanism that causes ER-resident proteins to relocate to the cytosol and was found to be constitutively active in cancer cells (44). Subcellular protein fractionation using minimal concentration of digitonin that results in proper separation of the different subcellular fractions was carried out in A549 and T47D cells subjected to ER stress. This was followed by an analysis of the localization of endogenous ER-resident proteins AGR2, PDIA3 along with the integral protein calnexin (Fig. 6*D* and supplemental Fig. S6*D*). In response to ER stress, we observed clear enrichment of AGR2 and slight increase of PDIA3 in cytosolic fraction indicating that both proteins are refluxed to the cytosol to some extent, which may reflect disperse delocalization observed by fluorescence microscopy. On the other hand, because of a relatively small proportion of AGR2 and PDIA3 detected in the cytosol compared with the total amount of proteins, the expansion of ER in response to stress stimuli as an integral part of the cellular program to overcome ER stress associated with increased formation of AGR2–PDIA3 complex has to be taken into account as well (45, 46, 47).

### Predicted Model for AGR2–PDIA3 Heterocomplex

The development of AGR2–PDIA3 heterocomplex was experimentally confirmed as shown in previous paragraphs. Nevertheless, although AGR2 protein can act as monomer or dimer (15, 28) and may develop even higher oligomeric structures (48), there is no evidence of AGR2–PDIA3 heterocomplex stoichiometry. For this reason, we prepared two models of this heterocomplex by *ab initio* docking on GalaxyHeteromer web server (http://galaxy.seoklab.org/cgi-bin/submit.cgi?type=HETEROMER; (33)). Similar heterocomplex *ab initio* models were prepared using ClusPro (34). All these docking simulations were performed in triplicates. In addition, the BADock server (38) was used to predict the binding interaction energy in between PDIA3 and AGR2 either in monomeric form or in dimeric form. Based on cluster population, both GalaxyHeteromer (supplemental Table S3) and ClusPro (supplemental Table S4) predicted the AGR2 monomer to be preferred over the dimer for the interaction with PDIA3. BADock, which predicts binding energies based on all docking solutions, also favors the monomer (−10.246 kcal/mol) over the dimer (−9.745 kcal/mol) by small margin. Interestingly, predictions for both monomer and dimer agreed on a preferred binding interface in between domains a and a' (supplemental Data File S3). Notably, this conformation not only is vastly preferred for AGR2 dimer but also is the most commonly observed for AGR2 monomer. An alternative interface is predicted in domain b or b' (supplemental Data Files S4 and S5), and few solutions were obtained where PDIA3 was bound to other parts of the protein (Table 3 and supplemental Data File S6). It is worth to mention here that b-domain predictions are consistent with previous observations indicating that this domain is responsible for protein binding (49). However, considering that GalaxyHeteromer results were consistent across replicates (supplemental Data Files S7 and S8) and with the other methods (ClusPro and BADock binding energy predictions [supplemental Data Files S9 and S10)], we refined one replicate of its results on Rosetta online server (ROSIE; https://rosie.graylab.jhu.edu/; (37)). Interestingly, the resulting interface score (supplemental Tables S5 and S6) agreed on the preferred monomeric binding along the a–a' interface.

**Table 3:** Binding interface classification of the top 10 solutions from monomer and dimer AGR2 docking experiments to PDIA3

Docking server	AGR2 quaternary structures	Total number of models (calculated from triplicates) for PDIA3 binding site			
		Between a a' domains	b domain	b' domain	Outside a and a' domains
GalaxyHeteromer	Monomer	21	3	3	3
	Dimer	15	6	0	9
ClusPro (balanced score)	Monomer	30	0	0	0
	Dimer	30	0	0	0

**Fig. 7:**
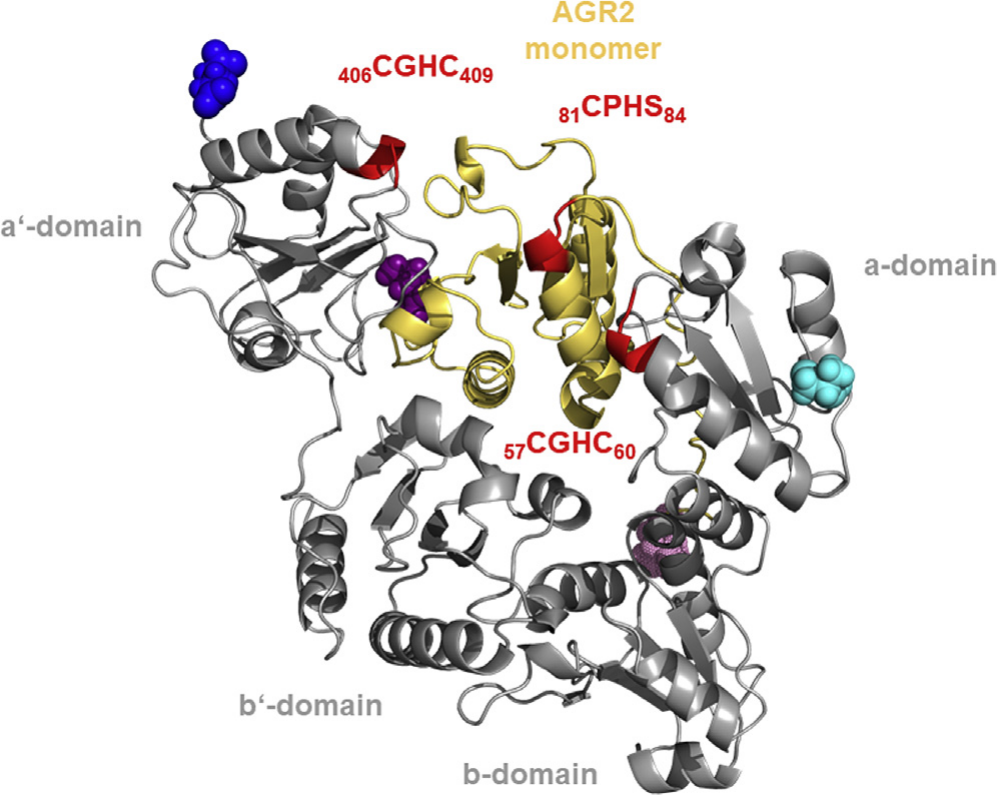
**Visualization of the interaction between AGR2 monomer and PDIA3.** A representative of the best docking solution from GalaxyHeteromer experiment for monomeric AGR2 (*yellow*) is visualized in complex with PDIA3 (*gray*). N termini (Ile36) and C termini (Leu175) of ARG2 are indicated in *light* and *purple* solid van der Waals radii spheres, respectively. N termini (Ser25) and C termini (Glu493) of PDIA3 are indicated in *light* and *dark blue solid van der Waals radii spheres*, respectively. PDIA3 domains are labeled according to the description of the PDB file (36) and following this legend: a-domain comprises residues Ser25–Gly133; b-domain comprises residues Pro134–Gly242; b'-domain comprises residues Ile243–Lys366; and a'-domain comprises residues Ser367–Ala484. Active site motifs are shown in *red* for both proteins; their sequences and residue numbers are indicated in proximity and with the same color code. AGR2, anterior gradient 2; PDB, Protein Data Bank; PDIA3, protein disulfide isomerase A3.

We chose as an illustrative example the best scoring monomer model from GalaxyHeteromer (Fig. 7) and observed that this interaction leads to blocking of both active-site motifs (CXXC) of PDIA3 (amino acids 57–60 and 406–409) that are located closely to the interaction interface, which includes 11 salt bridges, 24 hydrogen bonds, and 334 nonbonded contacts. In less probable model of dimer AGR2–PDIA3 heterocomplex, PDIA3 forms U-shaped structure to which AGR2 dimer binds. There are one salt bridge, two hydrogen bonds, and 58 nonbonded contacts on the interaction interface. Active motifs CXXS (amino acids 81–84 for AGR2) and CXXC (amino acids 406–409 for PDIA3) are located on the periphery of the interaction interface.

## Discussion

Correct protein folding remains the critical prerequisite for the mature protein structure and function. One of the key contributors involved in protein folding is the PDI family. Chaperone functions of PDIs are associated with the regulation of protein retention, secretion, quality control, ER-associated degradation, and maintenance of ER homeostasis (50). AGR2 as a PDI family member (14) was shown to be associated with the development of several diseases, including cancer (3). However, to date, hundreds of client proteins were predicted, and only a limited number of proteins were validated to interact and/or to be folded by AGR2. The first study by yeast two-hybrid screen identified several AGR2-interacting partners, however, without deeper characterization except for several surface molecules, including epithelial cell adhesion molecule, epidermal growth factor receptor, C4.4a, and mucin 2 (14, 16, 23, 51, 52, 53). Recently, a comprehensive PPI screen was reported by Tiemann *et al.* (54), identifying predominantly core components of Hippo and mammalian target of rapamycin complex signaling pathways and their downstream effectors as AGR2-interacting partners, and by Worfolk *et al.* (55), who identified AGR2 interaction with chaperones in redox-responsible and disulfide-dependent complexes. Comparing AGR2-interacting partners from these three studies ((54, 55) and ours), we identified the set of 14 overlapping proteins (Table 4 and supplemental Data File S11*A*). These proteins are mainly situated in ER and cytoplasm (supplemenatal Data File S11*B*) and play biological roles in protein folding process in ER and in responses to various stress stimuli in the cell and molecularly function as disulfide isomerases or oxidoreductases (supplemental Data File S11*C*; http://cpdb.molgen.mpg.de/). Interestingly, Tiemann's analysis (54) of three different cell lines resulted in the identification of 66 (in PANC-1 cells), 155 (in MIA PaCa-2 cells), and 365 proteins (in MCF7 cells) potentially interacting with AGR2, compared with our LC–MS/MS analysis, which identified 1055 interacting proteins (152 with log2FC > 0, *q* < 0.05) in T47D cells and 245 proteins (21 with log2FC > 0, *q* < 0.05) binding to AGR2 in H1299 cells. In both studies, there is a similar evidence that cells endogenously expressing AGR2 exhibit a substantially higher number of identified AGR2-interacting partners compared with cells with stable transfection of the *AGR2* coding sequence.

**Table 4:** AGR2-interacting proteins overlapped in three independent studies ((54, 55) and ours)

UniProt ID	Gene name	Protein abbreviation	Protein name
**O95994**	**AGR2**	**AGR2**	Anterior gradient 2
P06576	ATP5B	ATPB	ATP synthase, H+ transporting, mitochondrial F1 complex, beta polypeptide
P07237	P4HB	PDIA1	Prolyl 4-hydroxylase, beta polypeptide
P07355	ANXA2	ANXA2	Annexin A2
P10599	TXN	THIO	Thioredoxin
**P11021**	**HSPA5**	**GRP78**	**Heat shock 70 kDa protein 5 (glucose-regulated protein, 78 kDa)**
P23284	PPIB	PPIB	Peptidylprolyl isomerase B (cyclophilin B)
P27824	CANX	CALX	Calnexin
P30040	ERP29	ERP29	Endoplasmic reticulum protein 29
**P30101**	**PDIA3**	**PDIA3**	**Protein disulfide isomerase family A, member 3**
P40926	MDH2	MDHM	Malate dehydrogenase, mitochondrial
P62937	PPIA	PPIA	Peptidylprolyl isomerase A (cyclophilin A)
Q13162	PRDX4	PRDX4	Peroxiredoxin 4
**Q15084**	**PDIA6**	**PDIA6**	**Protein disulfide isomerase family A, member 6**

Proteins highlighted in bold are identified as overlapping proteins between T47D and H1299^AGR2^ cell lines in our study.

Among 15 proteins overlapping between T47D and H1299 cells in our study, 14 proteins including PDIA3 and PDIA6 were found by Tiemann *et al.* (54) and PDIA3 and PDIA6 also by Worfolk *et al.* (55) who, however, mainly focused on mammalian target of rapamycin–related aspects and on the oxidative stress response, respectively, and did not validate these interactions. It is also important to note that four independent studies (54, 55, 56), and ours, on nine cancer cell lines of different origin in total, have identified GRP78/BiP/HSPA5, the ER residential chaperone and one of key central regulators of ER processes (57), to interact with AGR2. Interestingly, this interaction was stronger under ER stress after TUN treatment in HCT-8 and HeLa cells (56). ER mammalian protein–protein interaction trap approach has also confirmed the interaction between AGR2 and two other ER residential proteins, endoplasmin (ENPL and HSP90B1) and RPN1, interacting with AGR2 during its dimerization in a negative and positive manner, respectively (15), that were also detected in our analysis.

Another important point is the cellular localization of AGR2 and its interacting partners, which may vary depending on cellular processes and responses to surrounding stimuli observed by us and others (4). Very recently, a phenomenon of ER-to-cytosol-signaling has been described, which explains the interaction of AGR2 also with proteins localized outside the ER (44). During the ER-to-cytosol-signaling, typical ER-resident soluble proteins, including AGR2, PDIA3, and others, can be in response to ER stress refluxed into the cytosol. These released ER proteins may gain new functions through selective interactions with other proteins and influence their cytosolic functions (44). In addition to that a well-known ER-associated chaperone CALR (identified by us and Tiemann *et al.*) has already been described to have different molecular functions depending on its localization (ER, nucleus, cytosol, cell surface, and extracellular matrix) described in bigger detail in excellent reviews (58, 59). Although, in our study, predominantly members of the PDI family were significantly represented among proteins identified to interact with AGR2, several non-ER client proteins were also found. One of them is heterogenous ribonucleoprotein U for which the interaction with AGR2 was described in A549 cell line linking AGR2 to regulation of gene expression on the post-transcriptional level (60). Moreover, ER-resident proteins may be in crosstalk with mitochondrial proteins through mitochondria-associated ER–membrane process, where membranes of these organelles are in physical contact. This facilitates Ca^2+^ ion exchange and maintains Ca^2+^ homeostasis. Stress-70 protein (mitochondrial), also known as 75 kDa glucose-regulated chaperone GRP75/HSPA9, plays active role in intermembrane complex formation and through this in intracellular signal transduction (61).

Following functional analysis of the whole AGR2 interactome revealed the involvement of these proteins in regulation of the protein processing, metabolic pathways, and in the maintenance of cellular homeostasis. Predominantly, PDIA3 and PDIA6 were convincingly identified as proteins interacting with intracellular AGR2 by the implementation of two independent approaches applied in two different cellular models. Interestingly, literature search for the last 10 years revealed that PDIA3 (also ERp57 or GRP58) along with AGR2 had attracted the most attention in relation to cancer research (62). However, only two articles mentioned the potential interactions or functional crosstalk between these two proteins, though both are localized in ER and were shown to participate in carcinogenesis and possess features of biomarkers. The interaction of AGR2 and PDIA3/6 was validated by several different methods *in vitro*. IP with specific AGR2 Ab and PLA confirmed the interaction of both PDIA3 and PDIA6 with AGR2, and immunofluorescence showed colocalization of these proteins around the nucleus in presumed ER space, which is in accordance with previously described subcellular localization of these proteins (28, 63, 64).

The secretion of AGR2 outside the cells is frequently observed (65); however, the mechanism remains not fully elucidated. Our experiments revealed that under mild ER stress conditions, the level of extracellular AGR2 was significantly increased in comparison with intracellular AGR2. In parallel, we also observed enforced AGR2–PDIA3 complex formation in response to ER stress, especially after TUN treatment, indicating some function of this protein complex in tumor cells exposed to ER stress. It is now well established that the majority of proteins in secretory pathways require glycosylation in order to achieve proper folding (66) including number of studies showing that exposure to TUN induces both enhanced protein complex formation and increased protein secretion (67, 68). However, whether AGR2–PDIA3 complex is directly involved in the regulation of enhanced AGR2 secretion remains unclear, since our data clearly show interaction of AGR2–PDIA3 in ER, which is enhanced in response to ER stress induction followed by increased secretion of AGR2 to both cytosol and extracellular space. Thus, we believe that investigating the role of the AGR2–PDIA3 interaction in cancer cells may contribute to elucidation of their function and highlight their potential in targeted cancer therapy in the future.

## Data Availability

The MS proteomics data have been deposited to the ProteomeXchange Consortium *via* the Proteomics Identifications (PRIDE) (69) partner repository with the dataset identifiers PXD012690 (LFQ experiment with T47D cells) and PXD012687 (SILAC experiment with H1299 cells).

## Supplemental data

This article contains supplemental data (36, 54, 55).

## Conflict of interest

The authors declare no competing interests.
