# Supplementary material for: Characterization of the AGR2 Interactome Uncovers New Players of Protein Disulfide Isomerase Network in Cancer Cells
Source: Mol Cell Proteomics. 2021 Dec 18;21(2):100188. doi: 10.1016/j.mcpro.2021.100188 (PMC8816719; doi:10.1016/j.mcpro.2021.100188)
Supplement: Supplemental Material [file mmc12.pdf]

Tab. S1: List of the overlapping AGR2 interacting partners in T47D and H1299-AGR2 cells (log2FC > 0, q < 0.05) with protein localization and their roles in cells. ER, endoplasmic reticulum.

	Uniprot ID	log2FC T47D	log2FC H1299-AGR2	Entry name	Gene name	Protein name	Localization in cell (source: uniprot.org)	Role in cell (source: uniprot.org)
**1**	O95994	0.3762	12.5140	AGR2	**AGR2**	anterior gradient 2	ER	Required for MUC2 post-transcriptional synthesis and secretion. May play a role in the production of mucus by intestinal cells (By similarity). Proto-oncogene that may play a role in cell migration, cell differentiation and cell growth. Promotes cell adhesion (PubMed:23274113).
**2**	P14625	3.6711	7.2929	ENPL	**HSP90B1**	heat shock protein 90 beta family member 1	ER	Molecular chaperone that functions in the processing and transport of secreted proteins (By similarity). When associated with CNPY3, required for proper folding of Toll-like receptors (By similarity). Functions in endoplasmic reticulum associated degradation (ERAD) (PubMed:18264092). Has ATPase activity (By similarity).
**3**	P11021	4.2576	6.0623	GRP78	**HSPA5**	heat shock protein family A (Hsp70) member 5	ER	Endoplasmic reticulum chaperone that plays a key role in protein folding and quality control in the endoplasmic reticulum lumen (PubMed:2294010, PubMed:23769672, PubMed:23990668, PubMed:28332555). Involved in the correct folding of proteins and degradation of misfolded proteins via its interaction with DNAJC10/ERdj5, probably to facilitate the release of DNAJC10/ERdj5 from its substrate (By similarity). Acts as a key repressor of the ERN1/IRE1-mediated unfolded protein response (UPR) (PubMed:1550958, PubMed:19538957). In the unstressed endoplasmic reticulum, recruited by DNAJB9/ERdj4 to the luminal region of ERN1/IRE1, leading to disrupt the dimerization of ERN1/IRE1, thereby inactivating ERN1/IRE1 (By similarity). Accumulation of misfolded protein in the endoplasmic reticulum causes release of HSPA5/BiP from ERN1/IRE1, allowing homodimerization and subsequent activation of ERN1/IRE1 (By similarity). Plays an auxiliary role in post-translational transport of small presecretory proteins across endoplasmic reticulum (ER). May function as an allosteric modulator for SEC61 channel-forming translocon complex, likely cooperating with SEC62 to enable the productive insertion of these precursors into SEC61 channel. Appears to specifically regulate translocation of precursors having inhibitory residues in their mature region that weaken channel gating.AMPylation was initially reported to take place at Ser-365 and Thr-366 in vitro, and promote activation of HSPA5/BiP (PubMed:25601083). However, it was later shown that AMPylation takes place at Thr-518 and leads to inactivation of HSPA5/BiP.
**4**	P38646	0.5211	5.8264	GRP75	**HSPA9**	heat shock protein family A (Hsp70) member 9	mitochondria	Chaperone protein which plays an important role in mitochondrial iron-sulfur cluster (ISC) biogenesis. Interacts with and stabilizes ISC cluster assembly proteins FXN, NFU1, NFS1 and ISCU (PubMed:26702583). Regulates erythropoiesis via stabilization of ISC assembly (PubMed:21123823, PubMed:26702583). May play a role in the control of cell proliferation and cellular aging (By similarity).

**5**	P14314	4.9090	5.2369	GLU2B	**PRKCSH**	protein kinase C substrate 80K-H	ER	Regulatory subunit of glucosidase II that cleaves sequentially the 2 innermost alpha-1,3-linked glucose residues from the Glc2Man9GlcNAc2 oligosaccharide precursor of immature glycoproteins (PubMed:10929008). Required for efficient PKD1/Polycystin-1 biogenesis and trafficking to the plasma membrane of the primary cilia (By similarity)
**6**	Q96RQ3	1.9626	5.1296	MCCA	**MCCC1**	methylcrotonoyl-CoA carboxylase 1	mitochondria	Biotin-attachment subunit of the 3-methylcrotonyl-CoA carboxylase, an enzyme that catalyzes the conversion of 3-methylcrotonyl-CoA to 3-methylglutaconyl-CoA, a critical step for leucine and isovaleric acid catabolism.
**7**	Q15084	5.6068	4.8100	PDIA6	**PDIA6**	protein disulfide isomerase family A member 6	ER, cell membrane, melanosome	May function as a chaperone that inhibits aggregation of misfolded proteins (PubMed:12204115). Negatively regulates the unfolded protein response (UPR) through binding to UPR sensors such as ERN1, which in turn inactivates ERN1 signaling (PubMed:24508390). May also regulate the UPR via the EIF2AK3 UPR sensor (PubMed:24508390). Plays a role in platelet aggregation and activation by agonists such as convulxin, collagen and thrombin (PubMed:15466936).
**8**	Q9HCC0	7.9606	4.2815	MCCB	**MCCC2**	methylcrotonoyl-CoA carboxylase 2	mitochondria	Carboxyltransferase subunit of the 3-methylcrotonyl-CoA carboxylase, an enzyme that catalyzes the conversion of 3-methylcrotonyl-CoA to 3-methylglutaconyl-CoA, a critical step for leucine and isovaleric acid catabolism.
**9**	P27797	6.0288	3.3646	CALR	**CALR**	calreticulin	ER	Calcium-binding chaperone that promotes folding, oligomeric assembly and quality control in the endoplasmic reticulum (ER) via the calreticulin/calnexin cycle. This lectin interacts transiently with almost all of the monoglucosylated glycoproteins that are synthesized in the ER. Interacts with the DNA-binding domain of NR3C1 and mediates its nuclear export. Involved in maternal gene expression regulation. May participate in oocyte maturation via the regulation of calcium homeostasis (By similarity).
**10**	P05165	1.1893	3.3443	PCCA	**PCCA**	propionyl-CoA carboxylase subunit alpha	mitochondria	This is one of the 2 subunits of the biotin-dependent propionyl-CoA carboxylase (PCC), a mitochondrial enzyme involved in the catabolism of odd chain fatty acids, branched-chain amino acids isoleucine, threonine, methionine, and valine and other metabolites (PubMed:8434582, PubMed:6765947). Propionyl-CoA carboxylase catalyzes the carboxylation of propionyl-CoA/propanoyl-CoA to D-methylmalonyl-CoA/(S)-methylmalonyl-CoA (PubMed:8434582, PubMed:6765947, PubMed:10101253). Within the holoenzyme, the alpha subunit catalyzes the ATP-dependent carboxylation of the biotin carried by the biotin carboxyl carrier (BCC) domain, while the beta subunit then transfers the carboxyl group from carboxylated biotin to propionyl-CoA (By similarity). Propionyl-CoA carboxylase also significantly acts on butyryl-CoA/butanoyl-CoA, which is converted to ethylmalonyl-CoA/(2S)-ethylmalonyl-CoA at a much lower rate (PubMed:6765947). Other alternative minor substrates include (2E)-butenoyl-CoA/crotonoyl-CoA (By similarity).
**11**	Q00839	0.8350	3.2388	HNRPU	**HNRNPU**	heterogeneous nuclear ribonucleoprotein U	nucleus	DNA- and RNA-binding protein involved in several cellular processes such as nuclear chromatin organization, telomere-length regulation, transcription, mRNA alternative splicing and stability, Xist-mediated transcriptional silencing and mitotic cell progression (PubMed:10490622, PubMed:18082603, PubMed:19029303, PubMed:22325991, PubMed:25986610, PubMed:28622508).

**12**	P30101	6.0095	2.6868	PDIA3	**PDIA3**	protein disulfide isomerase family A member 3	ER	
**13**	P12236	0.7001	2.4526	ADT3	**SLC25A6**	solute carrier family 25 member 6	mitochondria	Catalyzes the exchange of cytoplasmic ADP with mitochondrial ATP across the mitochondrial inner membrane. May participate in the formation of the permeability transition pore complex (PTPC) responsible for the release of mitochondrial products that triggers apoptosis.
**14**	P04843	1.0611	2.3978	RPN1	**RPN1**	ribophorin I	ER	Subunit of the oligosaccharyl transferase (OST) complex that catalyzes the initial transfer of a defined glycan (Glc3Man9GlcNAc2 in eukaryotes) from the lipid carrier dolichol-pyrophosphate to an asparagine residue within an Asn-X-Ser/Thr consensus motif in nascent polypeptide chains, the first step in protein N-glycosylation. N-glycosylation occurs cotranslationally and the complex associates with the Sec61 complex at the channel-forming translocon complex that mediates protein translocation across the endoplasmic reticulum (ER). All subunits are required for a maximal enzyme activity.
**15**	O75369	0.2854	2.1332	FLNB	**FLNB**	filamin B	cytoskeleton	Connects cell membrane constituents to the actin cytoskeleton. May promote orthogonal branching of actin filaments and links actin filaments to membrane glycoproteins. Anchors various transmembrane proteins to the actin cytoskeleton. Interaction with FLNA may allow neuroblast migration from the ventricular zone into the cortical plate. Various interactions and localizations of isoforms affect myotube morphology and myogenesis. Isoform 6 accelerates muscle differentiation in vitro.
**16**	P11498	0.6543	1.8363	PYC	**PC**	pyruvate carboxylase	mitochondria	Pyruvate carboxylase catalyzes a 2-step reaction, involving the ATP-dependent carboxylation of the covalently attached biotin in the first step and the transfer of the carboxyl group to pyruvate in the second. Catalyzes in a tissue specific manner, the initial reactions of glucose (liver, kidney) and lipid (adipose tissue, liver, brain) synthesis from pyruvate.

Tab. S2: Statistically significant BIOCARTA and REACTOME pathways (NOM p-value < 0.05) based on Gene Set Enrichment Analysis (GSEA) of enriched AGR2 protein-protein interacting partners (log2FC > 0) identified in T47D cells using LFQ and in AGR2 stably transfected H1299 cells using SILAC quantification. Pathways highlighted in bold (i) were enriched for proteins from both T47D and H1299 cells AND (ii) contain proteins from PDIA family. PDIA family proteins are highlighted in bold. No statistically significant pathways were identified for proteins from H1299 cells in BIOCARTA database.

	T47D cells (LFQ)	Core enriched proteins
BIOCARTA	VITCB_PATHWAY	**PDIA1**
	HIF_PATHWAY	**PDIA1**
	IL2RB_PATHWAY	PPIA
REACTOME	**T47D cells (LFQ)**	**Core enriched proteins**
	**DIABETES_PATHWAYS**	CALR, HYOU1, **PDIA6**, TPP1, GRP78, DJB11, ENPL, SPCS2, SSRA
	**UNFOLDED_PROTEIN_RESPONSE**	CALR, HYOU1, **PDIA6**, TPP1, GRP78, DJB11, ENPL, SSRA
	ANTIGEN_PRESENTATION_FOLDING_ASSEMBLY_AND_PEPTIDE_LOADING_OF_CLASS_I_MHC	CALR, **PDIA3**, GRP78, CALX
	CHYLOMICRON_MEDIATED_LIPID_TRANSPORT	**PDIA1**
	LIPOPROTEIN_METABOLISM	**PDIA1**
	CALNEXIN_CALRETICULIN_CYCLE	CALR, **PDIA3**, GLU2B, UGGG1, CALX
	ACTIVATION_OF_CHAPERONES_BY_ATF6_ALPHA	CALR, GRP78, ENPL
	N_GLYCAN_TRIMMING_IN_THE_ER_AND_CALNEXIN_CALRETICULIN_CYCLE	CALR, **PDIA3**, GLU2B, UGGG1, CALX
	ACTIVATION_OF_CHAPERONE_GENES_BY_XBP1S	HYOU1, **PDIA6**, TPP1, DJB11, SSRA
	ACTIVATION_OF_CHAPERONE_GENES_BY_ATF6_ALPHA	CALR, GRP78, ENPL
	CLASS_I_MHC_MEDIATED_ANTIGEN_PROCESSING_PRESENTATION	CALR, **PDIA3**, GRP78, CALX
	INFLUENZA_LIFE_CYCLE	CALR, TPR, CANX, RS7, CLTC
	**ASPARAGINE_N_LINKED_GLYCOSYLATION**	CALR, **PDIA3**, LMAN1, GLU2B, UGGG1, OST48, CALX
	IMMUNE_SYSTEM	CALR, **PDIA3**, TPR, GLU2B, GRP78, OST48, ENPL, CALX, CLH1, CDC42, CATD, RAC1, SC61B, CADH1, NU214, CATB, THIOM
	ER_PHAGOSOME_PATHWAY	CALR, **PDIA3**
	COLLAGEN_FORMATION	PPIB, **PDIA1**
	ANTIGEN_PROCESSING_CROSS_PRESENTATION	CALR, **PDIA3**
	CITRIC_ACID_CYCLE_TCA_CYCLE	MDHM, SUCA, SDHA, DLST, ACO2, CISY
	EXTRACELLULAR_MATRIX_ORGANIZATION	PPIB, **PDIA1**
	PYRUVATE_METABOLISM_AND_CITRIC_ACID_TCA_CYCLE	MDHM, PDHA1, SUCA, SDHA, ODO2, ACON, CISY
	**POST_TRANSLATIONAL_PROTEIN_MODIFICATION**	CALR, **PDIA3**, LMAN1, GLU2B, UGGG1, OST48, CALX
	BINDING_AND_ENTRY_OF_HIV_VIRION	PPIA
	EGFR_DOWNREGULATION	CLH1, CDC42
	GLUCOSE_METABOLISM	CMC2, MDHM, M2OM, TXTP, PCKGM
	GLUCONEOGENESIS	CMC2, MDHM, M2OM, TXTP, PCKGM
	PERK_REGULATED_GENE_EXPRESSION	GRP78
	AXON_GUIDANCE	RHOA, CLH1, CDC42, RAC1
	APOBEC3G_MEDIATED_RESISTANCE_TO_HIV1_INFECTION	PPIA
	**H1299 cells (SILAC)**	**Core enriched proteins**
	TRAFFICKING_AND_PROCESSING_OF_ENDOSOMAL_TLR	ENPL
	DESTABILIZATION_OF_MRNA_BY_AUF1_HNRNP_D0	HS71B, HSP7C
	**UNFOLDED_PROTEIN_RESPONSE**	ENPL, GRP78, **PDIA6**, CALR, LMNA
	MUSCLE_CONTRACTION	VIME, TPM3
	ACTIVATION_OF_CHAPERONE_GENES_BY_ATF6_ALPHA	ENPL, GRP78, CALR
	STRIATED_MUSCLE_CONTRACTION	VIME, TPM3
	ACTIVATION_OF_CHAPERONES_BY_ATF6_ALPHA	ENPL, GRP78, CALR
	PERK_REGULATED_GENE_EXPRESSION	GRP78
	**DIABETES_PATHWAYS**	ENPL, GRP78, **PDIA6**, CALR, LMNA
	BRANCHED_CHAIN_AMINO_ACID_CATABOLISM	MCCA, MCCB
	MITOCHONDRIAL_PROTEIN_IMPORT	GRP75, CH60, ATPB, ATPA, ADT3, TOMM22
	METABOLISM_OF_AMINO_ACIDS_AND_DERIVATIVES	MCCA, MCCB
	**POST_TRANSLATIONAL_PROTEIN_MODIFICATION**	GLU2B, CALR, **PDIA3**, OST48, RPN1, GANAB, DAD1
	**ASPARAGINE_N_LINKED_GLYCOSYLATION**	GLU2B, CALR, **PDIA3**, OST48, RPN1, GANAB, DAD1

NOM p-value: ● 0.00 ● 0.01 ● 0.02 ● 0.03 ● 0.04 ○ 0.05

enrichment score: ○ 1.0 ○ 0.9 ○ 0.8 ○ 0.7 ○ 0.6 ○ 0.5

Tab. S3: Binding interface classification of the top 10 solutions from Galaxy Heteromer of AGR2 monomeric or dimeric form to PDIA3. The first column identifies the binding interface. The second one the quaternary structure of AGR2. The third one the replica number. The fourth one the rank of the model, the fifth the score from Galaxy Heteromer (in arbitrary units) and the sixth the cluster size.

PDIA3 binding site	AGR2 quaternary structures	Replica	Model number (rank)	TongDock_A score [a.u.]	TongDock_A cluster size [N]
Between a, a' domains	Monomer	1	1	963.472	23
Between a, a' domains	Monomer	2	1	963.472	23
Between a, a' domains	Monomer	3	1	963.472	23
Between a, a' domains	Monomer	1	2	923.644	20
Between a, a' domains	Monomer	2	2	923.644	20
Between a, a' domains	Monomer	3	2	923.644	20
Between a, a' domains	Monomer	1	3	911.832	20
Between a, a' domains	Monomer	2	3	911.832	20
Between a, a' domains	Monomer	3	3	911.832	20
Between a, a' domains	Monomer	1	5	897.613	14
Between a, a' domains	Monomer	2	5	897.613	14
Between a, a' domains	Monomer	3	5	897.613	14
Between a, a' domains	Monomer	1	7	789.893	13
Between a, a' domains	Monomer	2	7	789.893	13
Between a, a' domains	Monomer	3	7	789.893	13
Between a, a' domains	Monomer	1	8	781.039	12
Between a, a' domains	Monomer	2	8	781.039	12
Between a, a' domains	Monomer	3	8	781.039	12
Between a, a' domains	Monomer	1	10	893.49	10
Between a, a' domains	Monomer	2	10	893.49	10
Between a, a' domains	Monomer	3	10	893.49	10
Between a, a' domains	Dimer	1	4	1130.624	11
Between a, a' domains	Dimer	2	4	1130.624	11
Between a, a' domains	Dimer	3	4	1130.624	11
Between a, a' domains	Dimer	1	5	944.027	11
Between a, a' domains	Dimer	2	5	944.027	11
Between a, a' domains	Dimer	3	5	944.027	11
Between a, a' domains	Dimer	1	6	869.312	11
Between a, a' domains	Dimer	2	6	869.312	11
Between a, a' domains	Dimer	3	6	869.312	11
Between a, a' domains	Dimer	1	8	1009.848	10
Between a, a' domains	Dimer	2	8	1009.848	10
Between a, a' domains	Dimer	3	8	1009.848	10
Between a, a' domains	Dimer	1	9	967.513	10
Between a, a' domains	Dimer	2	9	967.513	10
Between a, a' domains	Dimer	3	9	967.513	10
b domain	Monomer	1	6	907.039	13
b domain	Monomer	2	6	907.039	13
b domain	Monomer	3	6	907.039	13
b domain	Dimer	1	2	860.152	13
b domain	Dimer	2	2	860.152	13
b domain	Dimer	3	2	860.152	13
b domain	Dimer	1	7	829.141	11
b domain	Dimer	2	7	829.141	11
b domain	Dimer	3	7	829.141	11

b' domain	Monomer	1	9	989.408	10
b' domain	Monomer	2	9	989.408	10
b' domain	Monomer	3	9	989.408	10
Outside a, a' domains	Monomer	1	4	911.545	20
Outside a, a' domains	Monomer	2	4	911.545	20
Outside a, a' domains	Monomer	3	4	911.545	20
Outside a, a' domains	Dimer	1	1	920.692	14
Outside a, a' domains	Dimer	2	1	920.692	14
Outside a, a' domains	Dimer	3	1	920.692	14
Outside a, a' domains	Dimer	1	3	850.929	12
Outside a, a' domains	Dimer	2	3	850.929	12
Outside a, a' domains	Dimer	3	3	850.929	12
Outside a, a' domains	Dimer	1	10	877.029	10
Outside a, a' domains	Dimer	2	10	877.029	10
Outside a, a' domains	Dimer	3	10	877.029	10

Tab. S4: Binding interface classification of the top 10 solutions from ClusPro of AGR2 monomeric or dimeric form to PDIA3. The first column identifies the binding interface. The second one the quaternary structure of AGR2. The third one the replica number. The fourth one the rank of the model, the fifth the cluster size, and the sixth the score from ClusPro (using the balanced coefficient), in arbitrary units.

PDIA3 binding site	AGR2 quaternary structures	Replica	Cluster number (rank)	Cluster size	Weighted score of the cluster center [a.u.]
Between a, a' domains	Monomer	1	0	146	-916.6
Between a, a' domains	Monomer	2	0	146	-916.6
Between a, a' domains	Monomer	3	0	146	-916.6
Between a, a' domains	Monomer	1	1	44	-779.2
Between a, a' domains	Monomer	2	1	44	-779.2
Between a, a' domains	Monomer	3	1	44	-779.2
Between a, a' domains	Monomer	1	2	40	-815.7
Between a, a' domains	Monomer	2	2	40	-815.7
Between a, a' domains	Monomer	3	2	40	-815.7
Between a, a' domains	Monomer	1	3	39	-773.0
Between a, a' domains	Monomer	2	3	39	-773.0
Between a, a' domains	Monomer	3	3	39	-773.0
Between a, a' domains	Monomer	1	4	38	-764.9
Between a, a' domains	Monomer	2	4	38	-764.9
Between a, a' domains	Monomer	3	4	38	-764.9
Between a, a' domains	Monomer	1	5	32	-779.7
Between a, a' domains	Monomer	2	5	32	-779.7
Between a, a' domains	Monomer	3	5	32	-779.7
Between a, a' domains	Monomer	1	6	27	-839.2
Between a, a' domains	Monomer	2	6	27	-839.2
Between a, a' domains	Monomer	3	6	27	-839.2
Between a, a' domains	Monomer	1	7	26	-750.3
Between a, a' domains	Monomer	2	7	26	-750.3
Between a, a' domains	Monomer	3	7	26	-750.3
Between a, a' domains	Monomer	1	8	26	-769.3
Between a, a' domains	Monomer	2	8	26	-769.3
Between a, a' domains	Monomer	3	8	26	-769.3
Between a, a' domains	Monomer	1	9	26	-775.6
Between a, a' domains	Monomer	2	9	26	-775.6
Between a, a' domains	Monomer	3	9	26	-775.6
Between a, a' domains	Dimer	1	0	48	-1004.1
Between a, a' domains	Dimer	2	0	48	-1004.1
Between a, a' domains	Dimer	3	0	48	-1004.1
Between a, a' domains	Dimer	1	1	42	-967.5
Between a, a' domains	Dimer	2	1	42	-967.5
Between a, a' domains	Dimer	3	1	42	-967.5
Between a, a' domains	Dimer	1	2	37	-850.7
Between a, a' domains	Dimer	2	2	37	-850.7
Between a, a' domains	Dimer	3	2	37	-850.7
Between a, a' domains	Dimer	1	3	32	-869.5
Between a, a' domains	Dimer	2	3	32	-869.5
Between a, a' domains	Dimer	3	3	32	-869.5
Between a, a' domains	Dimer	1	4	31	-855.9
Between a, a' domains	Dimer	2	4	31	-855.9
Between a, a' domains	Dimer	3	4	31	-855.9

Between a, a' domains	Dimer	1	5	30	-867.8
Between a, a' domains	Dimer	2	5	30	-867.8
Between a, a' domains	Dimer	3	5	30	-867.8
Between a, a' domains	Dimer	1	6	26	-816.8
Between a, a' domains	Dimer	2	6	26	-816.8
Between a, a' domains	Dimer	3	6	26	-816.8
Between a, a' domains	Dimer	1	7	26	-830.4
Between a, a' domains	Dimer	2	7	26	-830.4
Between a, a' domains	Dimer	3	7	26	-830.4
Between a, a' domains	Dimer	1	8	23	-832.4
Between a, a' domains	Dimer	2	8	23	-832.4
Between a, a' domains	Dimer	3	8	23	-832.4
Between a, a' domains	Dimer	1	9	23	-808.1
Between a, a' domains	Dimer	2	9	23	-808.1
Between a, a' domains	Dimer	3	9	23	-808.1

Tab. S5: Statistics describing the lowest-interface-energy structures of monomer AGR2-PDIA3 complex generated by Rosetta online server.

Decoy	1	2	3	4	5	6	7	8	9	10
Total score [a.u.]	-568.341	-568.301	-568.150	-568.019	-567.959	-567.955	-567.876	-567.850	-567.830	-567.829
RMSD [Å]	0.703	0.696	0.7	0.711	0.701	0.692	0.702	0.657	0.659	0.659
Interface score [a.u.]	-25.705	-25.494	-25.772	-25.421	-25.911	-25.748	-25.728	-25.681	-25.517	-25.517
Irms [Å]	0.251	0.252	0.246	0.249	0.249	0.254	0.246	0.238	0.241	0.242
Van der Waals attractive energy [kcal/mol]	-971.089	-970.761	-970.476	-969.692	-970.086	-970.522	-969.701	-970.016	-969.876	-969.876
Side-chain conformational energy [kcal/mol]	40.91	40.902	40.863	40.909	40.806	40.743	40.85	40.87	40.954	40.954
Electrostatic energy [kcal/mol]	-6.857	-6.917	-6.922	-6.838	-6.917	-6.842	-6.905	-6.876	-6.844	-6.844
Pair-wise interaction energy [kcal/mol]	-21.289	-21.464	-21.42	-21.215	-21.517	-21.347	-21.335	-21.525	-21.243	-21.243
Van der Waals repulsive energy [kcal/mol]	33.62	33.652	33.578	33.517	33.546	33.703	33.571	33.556	33.472	33.472
Solvation energy [kcal/mol]	418.384	418.443	418.198	417.307	418.053	418.14	417.562	417.8	417.322	417.322

Tab. S6: Statistics describing the lowest-interface-energy structures of dimer AGR2-PDIA3 complex generated by Rosetta online server.

Decoy	1	2	3	4	5	6	7	8	9	10
Total score [a.u.]	-584.642	-584.381	-584.369	-584.365	-584.039	-584.022	-583.462	-583.232	-582.950	-582.935
RMSD [Å]	2.452	2.569	2.677	2.346	2.433	2.702	2.776	3.632	2.556	3.067
Interface score [a.u.]	-7.215	-7.097	-6.787	-7.174	-7.309	-6.442	-6.314	-6.563	-5.587	-5.633
Irms [Å]	1.217	1.313	1.173	1.099	1.119	1.241	1.278	1.349	1.056	1.234
Van der Waals attractive energy [kcal/mol]	-1082.204	-1082.136	-1081.417	-1082.816	-1081.797	-1080.617	-1080.334	-1079.975	-1081.692	-1081.373
Side-chain conformational energy [kcal/mol]	56.399	56.386	56.27	56.598	56.368	56.136	56.351	56.286	56.369	56.438
Electrostatic energy [kcal/mol]	-6.991	-6.986	-6.932	-6.936	-6.925	-6.978	-6.958	-6.995	-6.911	-7.018
Pair-wise interaction energy [kcal/mol]	-19.577	-19.364	-19.408	-19.565	-19.416	-19.73	-19.422	-19.563	-18.956	-19.282
Van der Waals repulsive energy [kcal/mol]	78.201	78.095	78.197	78.505	78.171	78.657	78.125	78.32	78.482	78.084
Solvation energy [kcal/mol]	450.919	450.963	450.269	451.303	450.952	449.846	450.093	449.844	450.987	451.463

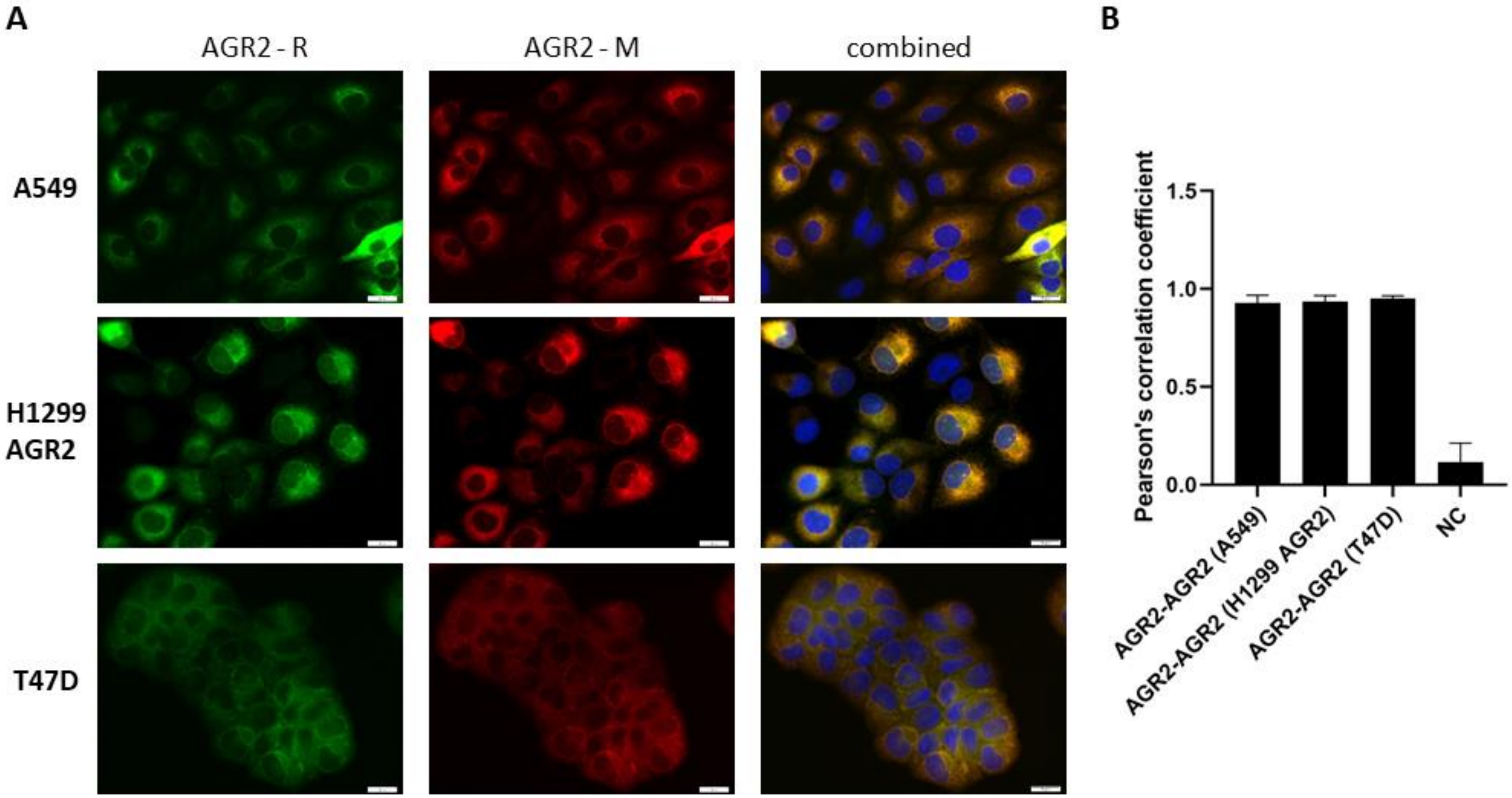

Fig. S1 (related to Fig. 3): Immunofluorescence staining of AGR2 in various cell lines and its quantification. (A) Various cell lines were stained with two different anti-AGR2 antibodies: a rabbit polyclonal from Abcam (AGR2– R) and a mouse monoclonal from Abnova (AGR2-M) labelled by Alexa Fluor 532 goat anti-rabbit IgG and Alexa Fluor 488 (both Abcam), respectively, and served as positive control. Cell nuclei (blue) were stained by Hoechst 33342. Scale bar is 20 µm. **(B)** Quantification of positive and negative controls using Fiji software.

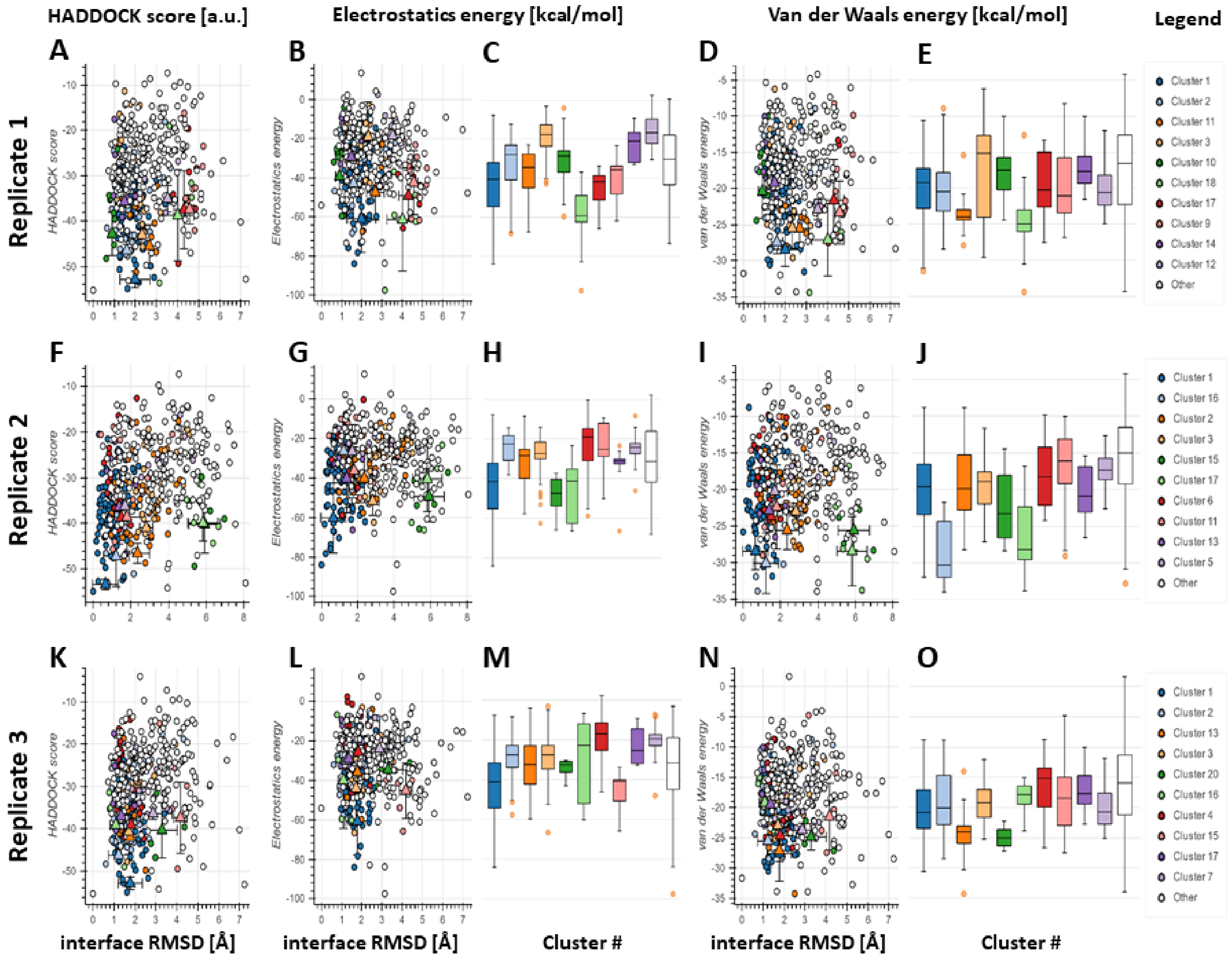

Fig. S2: Comparative analysis of AGR2-E7 peptide docking results across triplicates. Rows (A-E, F-J, and K-L) show results for individual replicates. The first column (A, F, K) shows the HADDOCK score for each individual docking pose and for the obtained clusters (with their associated dispersion measures) as a function of the interface RMSD. The second column (B, G, L) shows the Electrostatics energy term as a function of the interface RMSD. The third column (C, H, M) provides detail on the dispersion of the

obtained clusters on this Electrostatics energy metric. The fourth column (D, I, N) shows the van der Waals energy term as a function of the interface RMSD. The fifth column (E, J, O) provides detail on the dispersion of the obtained clusters on this van der Waals energy metric. To the right, a legend identifying the clusters in each replicate.

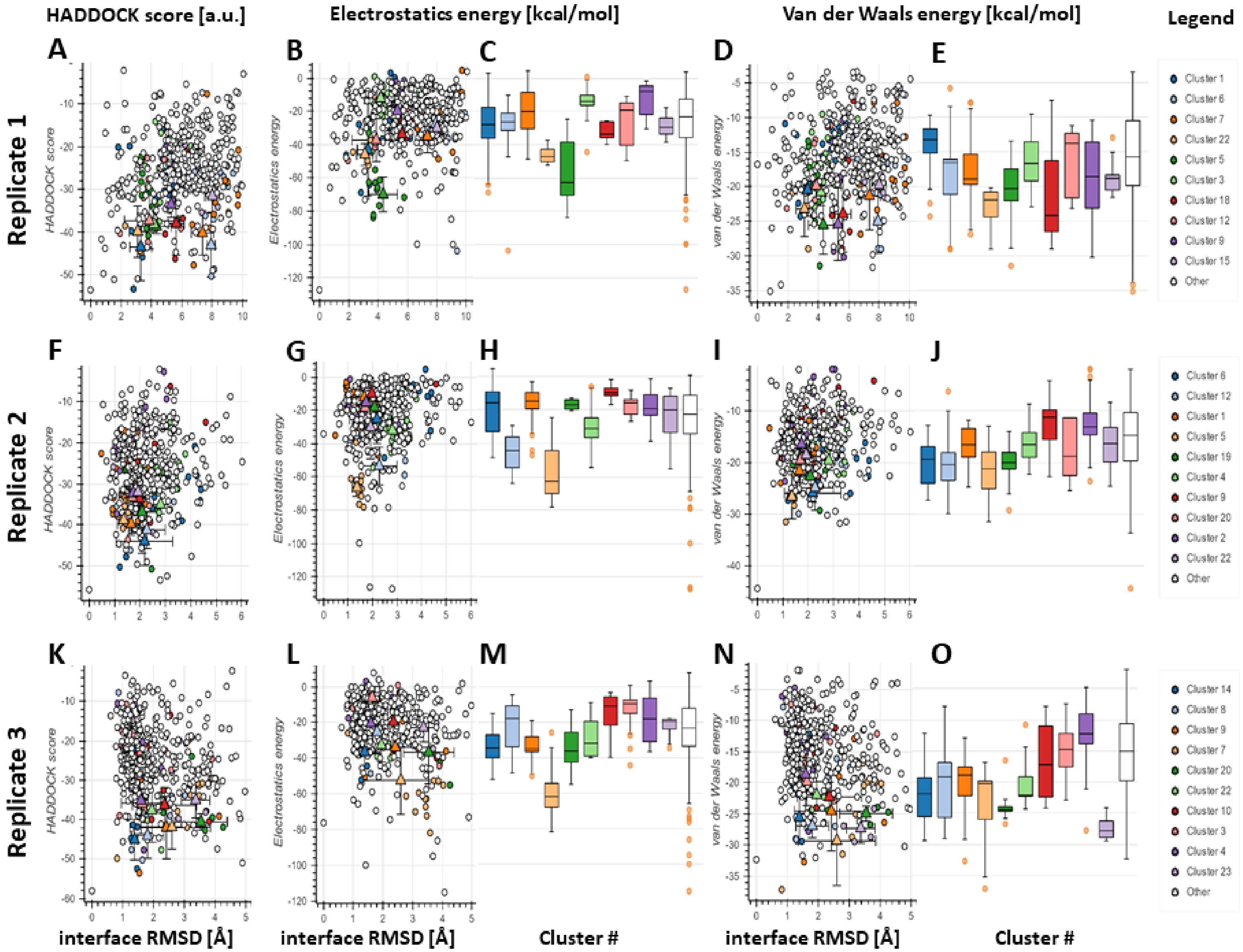

Fig. S3: Comparative analysis of AGR2-F4 peptide docking results across triplicates. Rows (A-E, F-J, and K-L) show results for individual replicates. The first column (A, F, K) shows the HADDOCK score for each individual docking pose and for the obtained clusters (with their associated dispersion measures) as a function of the interface RMSD.

The second column (B, G, L) shows the Electrostatics energy term as a function of the interface RMSD. The third column (C, H, M) provides detail on the dispersion of the obtained clusters on this Electrostatics energy metric. The fourth column (D, I, N) shows the van der Waals energy term as a function of the interface RMSD. The fifth column (E, J, O) provides detail on the dispersion of the obtained clusters on this van der Waals energy metric. To the right, a legend identifying the clusters in each replicate.

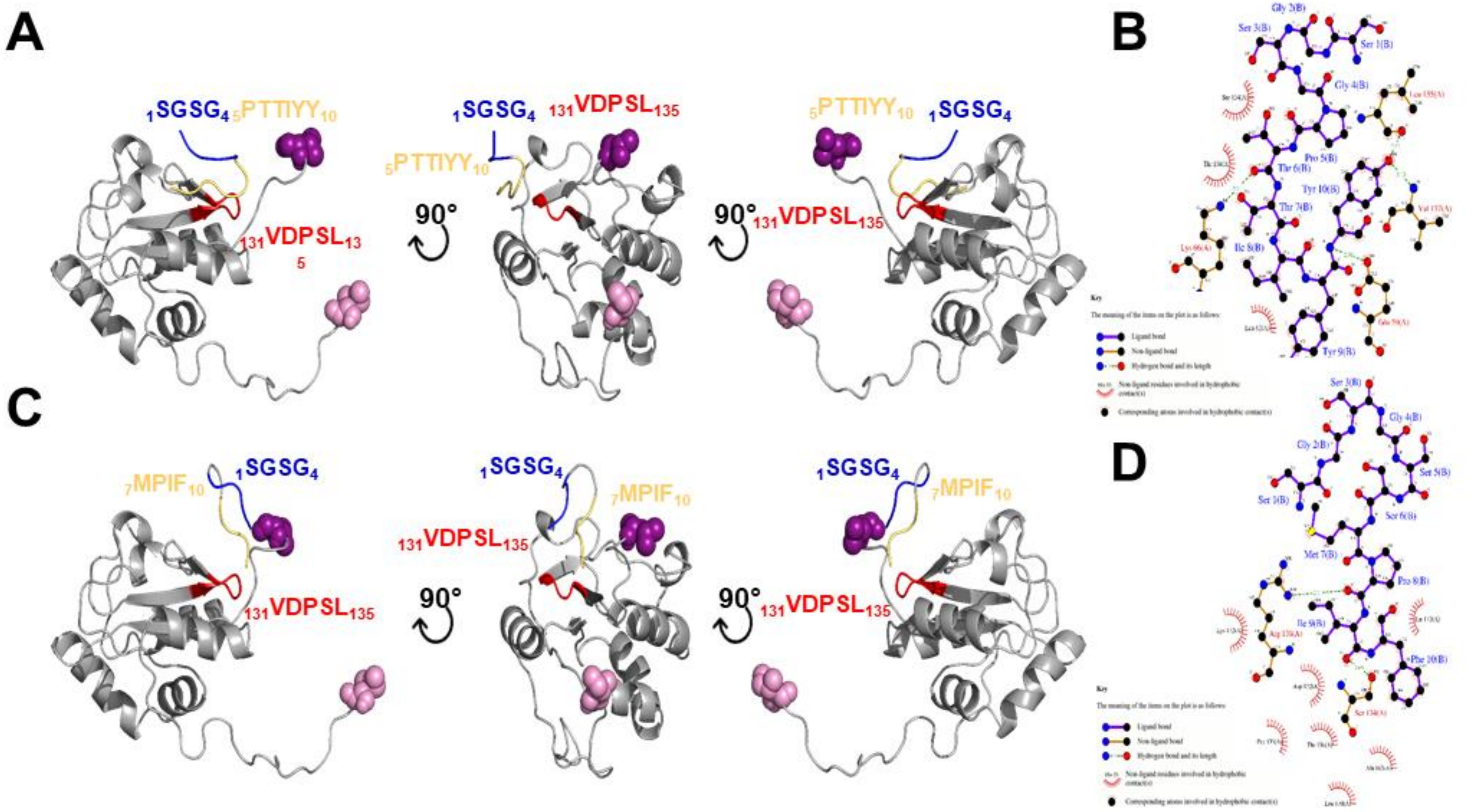

Fig. S4: Graphical summary of E7 and F4 peptides docking to AGR2. PyMOL software (The PyMOL Molecular Graphics System, Version 2.0 Schrödinger, LLC) was used for visualization of structures. PDBsum web server (http://www.ebi.ac.uk/thornton-srv/databases/pdbsum/Generate.html; (36)) was used for visual inspection of generated models to show interface interactions. The best scored model from the top ranked cluster has been chosen for inspection (**A**). Best AGR2-E7 peptide docking result. Binding residues from AGR2 highlighted in red, N-terminal motif of the peptide in blue, and C-terminal motif in yellow. N- and C-termini of AGR2 highlighted in light and dark purple

solid van der Waals radii spheres, respectively. (**B**) Interactions near the interface in between AGR2 and E7 peptide predicted by PDBsum. (**C**). Best AGR2-F4 peptide docking result. Details as in (A). (**D**) Interactions near the interface in between AGR2 and F4 peptide predicted by PDBsum.

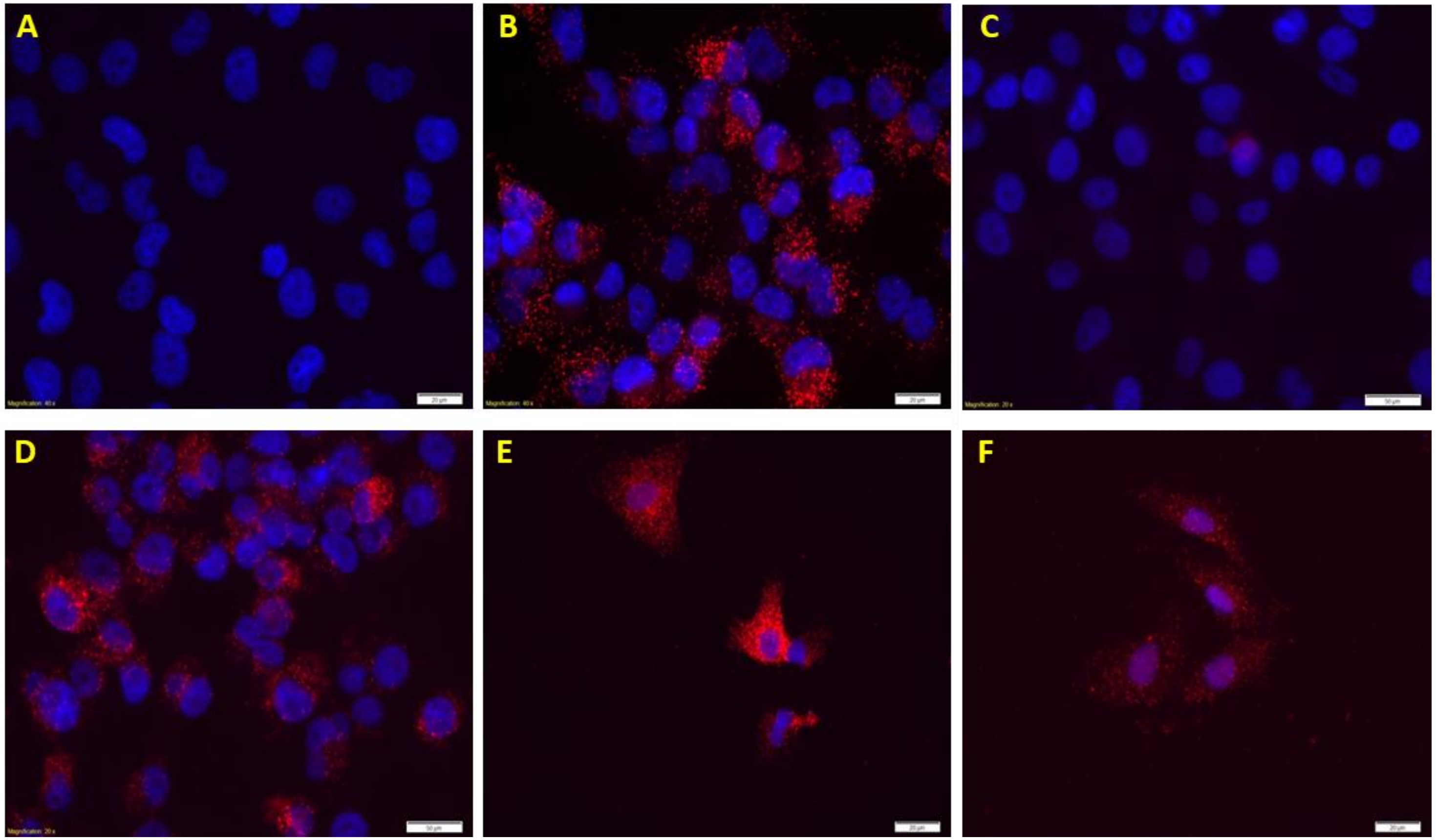

Fig. S5: Proximity ligation assay (PLA) was used to confirm interaction of AGR2 with PDIA3 and PDIA6 in different cell lines (related to Fig. 4). PLA for: (**A**) AGR2 and PDIA3 in H1299 cells (negative control), (**B**) AGR2 and PDIA3 in H1299-AGR2 cells, (**C**) AGR2 and PDIA6 in H1299 cells (negative control), (**D**) AGR2 and PDIA6 in H1299-AGR2 cells, (**E**) AGR2 and PDIA3 in A549 cells and (**F**) AGR2 and PDIA6 in A549 cells. Cell nuclei (blue) were stained by DAPI. Scale bar is 20 µm.

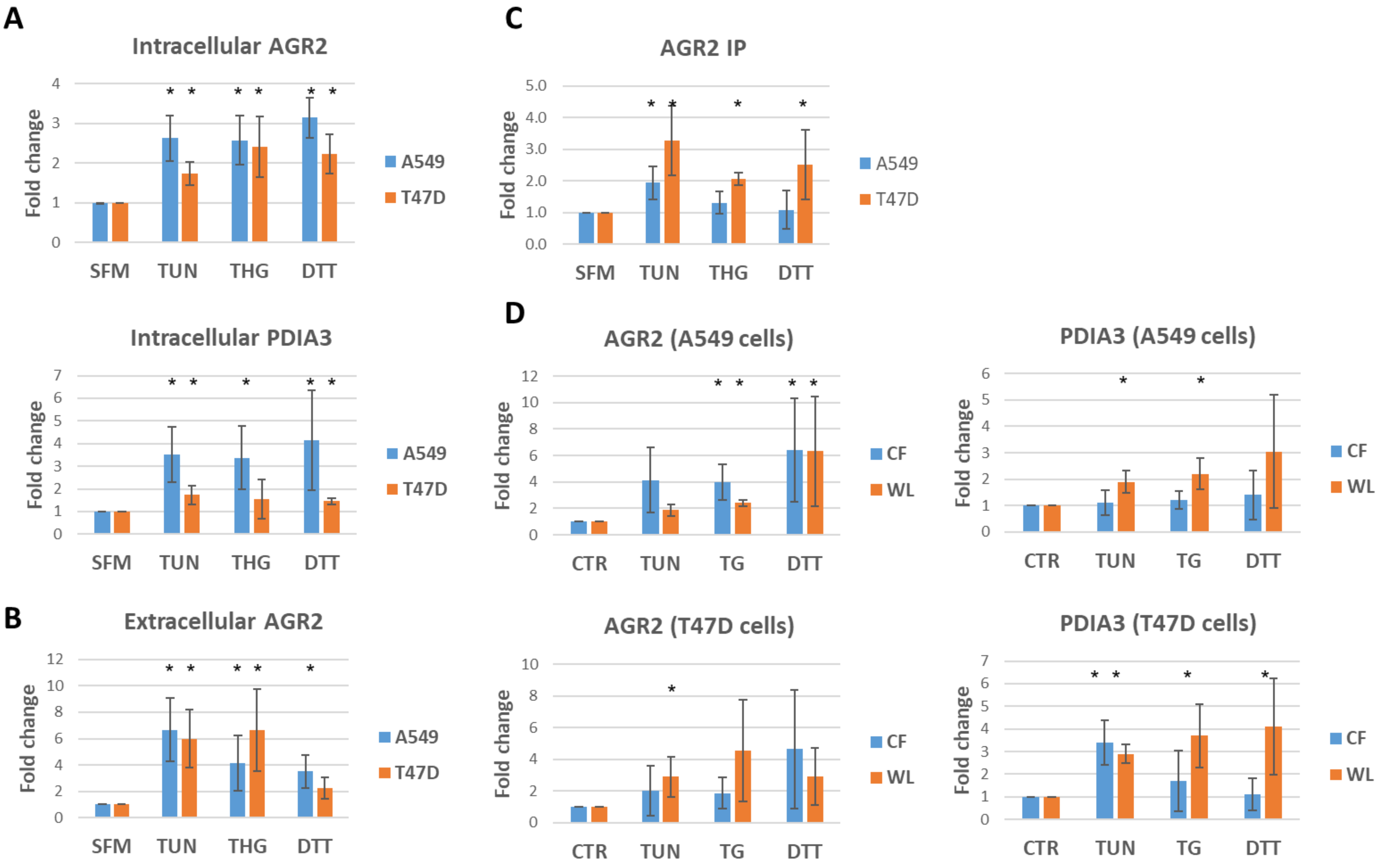

A
Intracellular AGR2
Fold change
SFM
TUN
THG
DTT
A549
T47D
Intracellular PDIA3
B
Extracellular AGR2
C
AGR2 IP
D
AGR2 (A549 cells)
PDIA3 (A549 cells)
AGR2 (T47D cells)
PDIA3 (T47D cells)
CTR
TG
CF
WL

Fig. S6 (on the previous page): Quantification of the protein levels in T47D and A549 cell lines (related to Fig. 6). Data were obtained from three independent biological experiments as a mean ± SD, where * indicates ($p \leq 0.05$, One-way ANOVA). (**A**) AGR2 and PDIA3 levels in whole cell lysates, (**B**) extracellular AGR2, (**C**) AGR2-PDIA3 complex level, (**D**) AGR2 and PDIA3 levels in cytosolic fraction (CF) and whole cell lysate (WL).

Legends of Data files

Data file S1: Mass spectrometry protein and peptide level data for identification of potential AGR2 interacting partners in T47D cells using label-free quantitation. Legend: expI=cells with DSP treatment, expC=cells without DSP treatment.

Data file S2: Mass spectrometry protein and peptide level data for identification and verification of potential AGR2 interacting partners in H1299 cells using SILAC quantitation.

Data file S3: **Docking solutions in binding site in between a and a' domains**. Pymol session containing all solutions in the top10 ranking doking poses from GalaxyHeteromer and ClusPro triplicate experiments using AGR2 monomer or dimer and PDIA3 that resulted in binding in between a and a' domains. The different pymol objects are identifed as follows MM_Q_R_C, where MM stands for Method, Q for quaternary structure, R for replica and C for cluster number (rank). The method legend is GH for Galaxy Heteromer or CP for ClusPro. The quaternary structure legend applies to AGR2 and is M for its monomeric state and D for its dimeric one. The replica number ranges in between 1 and 3. The lower cluster number indicates a better ranking in the docking experiment.

Data file S4: **Docking solutions in b-domain binding site**. Pymol session containing all solutions in the top10 ranking doking poses from GalaxyHeteromer and ClusPro triplicate experiments using AGR2 monomer or dimer and PDIA3 that resulted in binding the b-domain. Legends as in **Data file S3**.

Data file S5: **Docking solutions in b'-domain binding site**. Pymol session containing all solutions in the top10 ranking doking poses from GalaxyHeteromer and ClusPro triplicate experiments using AGR2 monomer or dimer and PDIA3 that resulted in binding the b'-domain. Legends as in **Data file S3**.

Data file S6: **Docking solutions in other binding sites**. Pymol session containing all solutions in the top10 ranking doking poses from GalaxyHeteromer and ClusPro triplicate experiments using AGR2 monomer or dimer and PDIA3 that resulted in other binding sites. Legends as in **Data file S3**.

Data file S7: **Docking solutions for Galaxy Heteromer AGR2monomer-PDIA3 triplicate experiment.** Pymol session containing all solutions in the top10 ranking doking poses from GalaxyHeteromer triplicate experiments using AGR2 monomer and PDIA3. Legends as in **Data file S3**.

Data file S8: **Docking solutions for Galaxy Heteromer AGR2dimer-PDIA3 triplicate experiment.** Pymol session containing all solutions in the top10 ranking doking poses from GalaxyHeteromer triplicate experiments using AGR2 dimer and PDIA3. Legends as in **Data file S3**.

Data file S9: **Docking solutions for Galaxy Heteromer AGR2monomer-PDIA3 triplicate experiment.** Pymol session containing all solutions in the top10 ranking doking poses from ClusPro triplicate experiments using AGR2 monomer and PDIA3. Legends as in **Data file S3**.

Data file S10: **Docking solutions for Galaxy Heteromer AGR2dimer-PDIA3 triplicate experiment.** Pymol session containing all solutions in the top10 ranking doking poses from ClusPro triplicate experiments using AGR2 dimer and PDIA3. Legends as in **Data file S3**.

Data file S11: List of AGR2 interactors presented in three independent studies. Annotation and gene ontology analysis of AGR2 interactors overlapping in three independent studies.
